# Multicomponent stress-strength reliability analysis using the inverted exponentiated Rayleigh distribution under block adaptive type-II progressive hybrid censoring and k-records

**DOI:** 10.1038/s41598-025-30570-9

**Published:** 2025-12-15

**Authors:** Haidy A. Newer

**Affiliations:** Department of Mathematics, Faculty of Education, Ain Shams University, Cairo, 11511 Egypt

**Keywords:** Multicomponent stress-strength reliability, Inverted Exponentiated Rayleigh distribution, Block Adaptive Type-II Progressive Hybrid Censoring, Upper k-records given inter-k-record times, Tierney and Kadane approximation, Metropolis-Hastings algorithm, Credible interval, Engineering, Mathematics and computing

## Abstract

We propose a statistical model for multicomponent stress-strength reliability under the inverted exponentiated Rayleigh distribution. The model is specifically designed for complex data structures where component strength is measured using block adaptive Type-II progressive hybrid censoring, while operational stress is captured as upper k-records with inter-k-record times. After formulating the reliability function for an *s*-out-of-*k* system, we develop both frequentist and Bayesian estimation procedures. Frequentist inference is based on the maximum likelihood estimator, from which we construct asymptotic and bootstrap confidence intervals. For the Bayesian analysis, we use squared error and linear exponential loss functions, obtaining estimates via the Tierney and Kadane approximation and a Metropolis-Hastings sampling algorithm. The performance of the estimators is evaluated through Monte Carlo simulations, which compare their bias and mean squared error. The results indicate that the Bayesian estimators are consistently more accurate than their frequentist counterparts. An analysis of two real datasets confirms the model’s practical utility for assessing system reliability in complex scenarios.

## Introduction

Reliability theory offers essential statistical methods for predicting the lifespan and performance of systems, a critical task for preventing failures and ensuring safety in fields like manufacturing and healthcare (Breneman et al.^1^). A central idea in this field is stress-strength reliability (SSR), which calculates the probability ($\zeta$) that a component’s strength (*X*) can withstand the operational stress (*Y*) it encounters, defined as $\zeta = P(X > Y)$ (Kotz et al.^2^). An accurate SSR assessment is fundamental for guaranteeing that components operate dependably under diverse conditions.

However, modern systems are rarely single components; they are complex assemblies of interconnected parts. This reality pushes the field toward multicomponent stress-strength reliability (MSSR), a more intricate and analytically demanding area (Khan and Jan^3^). Because the failure of one part can trigger a cascade across the entire system, precise MSSR analysis is indispensable for robust design and effective maintenance (Hassan et al.^4^). Researchers have extensively studied SSR and MSSR estimation using various distributions and data types (e.g., Baklizi^5^; Condino et al.^6^; Mahmoud et al.^7^; Nadar and Kizilaslan^8^; Tarvirdizade and Ahmadpour^9^; Wang and Ye^10^).

Modeling these phenomena correctly hinges on the right probability distribution. The inverted exponentiated Rayleigh (IER) distribution has become prominent for its impressive flexibility, capable of describing a wide range of hazard rate shapes–including increasing, decreasing, and bathtub curves–often seen in reliability testing (Fan and Gui^11^). This adaptability makes the IER distribution a strong candidate for modeling both stress and strength variables, providing a solid foundation for complex reliability analysis.

In practice, collecting complete lifetime data is often impossible due to high costs, time constraints, or the need to remove units from testing prematurely. This has led to the development of various censoring schemes (Balakrishnan^12^). Advanced methods like progressive censoring allow for the removal of surviving units at different stages, which enhances experimental flexibility (Balakrishnan and Cramer^13^). An even more sophisticated approach is the Type-II progressive hybrid censoring (AII-PHCS) scheme, which balances test duration and data yield by combining a fixed number of required failures with a time limit (Balakrishnan and Kundu^14^).

The AII-PHCS framework, introduced by Ng et al.^15^, allows an experiment to run beyond a preset time limit (*T*) to ensure a target number of failures (*m*) is observed. In a test with *n* units and a censoring plan $(R_1, \ldots , R_m)$, $R_i$ units are removed at the time of the *i*-th failure, $x_i$. The final removal group is $R_m = n - m - \sum _{i=1}^{m-1}R_i$. The scheme’s brilliance lies in its adaptive nature. If the *m*-th failure ($x_m$) happens before time *T*, the experiment concludes as a standard progressive Type-II test. However, if an earlier failure $x_J$ occurs before *T* but the next, $x_{J+1}$, is projected to occur after *T* (i.e., $x_J< T < x_{J+1}$), the plan adapts: all scheduled removals from $R_{J+1}$ to $R_{m-1}$ are canceled. Instead, all survivors are removed once the target *m*-th failure is finally observed. This redefines the final removal group as $R^*_{m} = n - m - \sum _{j=1}^{J} R_{j}$ and guarantees the desired number of failure observations. This practical flexibility has made adaptive censoring a subject of significant research interest (Dutta et al.^16^, Elshahhat et al.^17^, Nassar and Abo-Kasem^18^).

A further practical challenge is the inability to monitor all units simultaneously due to facility or equipment limitations. Block censoring addresses this by dividing the total *N* units into *g* smaller, independent groups (with $n_i$ units each, where $\sum n_i = N$). These blocks are then tested in parallel, making experiments more manageable and efficient when resources are constrained (Zhu^19^).

For stress modeling, extreme events are often the trigger for failure. This is where record statistics become invaluable for analysis in fields from industry to finance (Arnold et al.^20^; Safariyan et al.^21^; Singh and Singh^22^). While classical records track new maximums or minimums, *k*-records generalize this to the *k*-th largest or smallest value. An innovative extension known as upper *k*-records given inter-*k*-record times (URIT) captures not only the extreme values but also the crucial time dimension between them, providing richer data for processes driven by extremes (Hassan et al.^4^). The value of including inter-record times for improving reliability estimates has been recently highlighted (Pak et al.^23^).

These advanced tools for data collection–complex censoring for strength and record values for stress–have largely evolved separately. A critical gap exists in the literature: no unified framework can handle the complex but common scenario where component strength is measured using block adaptive Type-II progressive hybrid censoring (BAIIPH) while system stress follows the patterns of extreme records (URIT), particularly under the flexible IER distribution. This study bridges that critical gap by developing a novel and comprehensive statistical inference framework.

Our work makes several key contributions. We introduce and formulate a novel MSSR model that integrates BAIIPH for strength data and URIT for stress data, a combination that to our knowledge has not been explored. Building on this model, we develop a complete inferential toolkit using both frequentist and Bayesian methods. For the frequentist approach, we derive maximum likelihood estimators (MLEs) and construct asymptotic (ACI) and bootstrap-based confidence intervals. For the Bayesian paradigm, we employ squared error (SE) and linear exponential (LINEX) loss functions, deriving estimates using both Tierney and Kadane’s (TK) approximation and the Metropolis-Hastings (MH) algorithm. We then test the performance of all proposed estimators through extensive Monte Carlo simulations, evaluating bias, mean squared error, and interval properties. Finally, we demonstrate the model’s practical value by applying it to a real-world engineering dataset, offering tangible insights into system reliability.

The structure of this paper follows the logical progression of our analysis. We begin by formulating the fundamental multicomponent stress-strength reliability model, which integrates the flexible inverted exponentiated Rayleigh distribution with our specialized data collection schemes (Sect. “Data description and model formulation”). Building upon this model, we then construct a complete inferential toolkit, developing both frequentist and Bayesian estimation procedures (Sect. “Estimation of multicomponent stress-strength reliability”). The finite-sample performance of these proposed estimators is rigorously assessed through extensive Monte Carlo simulations (Sect. “Simulation study”). Finally, we demonstrate the framework's practical utility by applying it to two real-world engineering datasets (Sect. “Empirical applications”), before summarizing our conclusions and their broader significance (Sect. “Concluding remarks”).

## Data description and model formulation

Our analysis is built upon the inverted exponentiated Rayleigh distribution, a flexible lifetime model we use for both stress and strength variables. Since its introduction by Fatima and Ahmad^24^, the IER has become increasingly important in reliability analysis. Its key advantage is the ability to capture non-monotone hazard rate functions–including increasing, decreasing, and bathtub shapes–that appear frequently in real-world component failure data. This adaptability makes it a powerful alternative to more conventional distributions.

The IER distribution is part of the inverse exponentiated family and can be viewed as a special case of the inverted exponentiated Weibull distribution^25^. A random variable *X* following the IER distribution, denoted $X \sim \text {IER}(\theta , \lambda )$, has a shape parameter $\theta > 0$ and a scale parameter $\lambda > 0$. Its probability density function (PDF) is given by:

2.1 $$\begin{aligned} f(x; \theta , \lambda ) = 2\theta \lambda ^2 x^{-3} e^{-\lambda ^2/x^2} \left( 1 - e^{-\lambda ^2/x^2}\right) ^{\theta -1}, \quad x > 0. \end{aligned}$$

The corresponding cumulative distribution function (CDF) is:

2.2 $$\begin{aligned} F(x; \theta , \lambda ) = 1 - \left( 1 - e^{-\lambda ^2/x^2}\right) ^{\theta }, \quad x > 0. \end{aligned}$$

From these, we can define the reliability function *S*(*x*) and the hazard rate function (HRF) *h*(*x*) as:

2.3 $$\begin{aligned} S(x; \theta , \lambda ) = \left( 1 - e^{-\lambda ^2/x^2}\right) ^{\theta }, \end{aligned}$$

2.4 $$\begin{aligned} h(x; \theta , \lambda ) = \frac{f(x; \theta , \lambda )}{S(x; \theta , \lambda )} = \frac{2\theta \lambda ^2 x^{-3} e^{-\lambda ^2/x^2}}{1 - e^{-\lambda ^2/x^2}}. \end{aligned}$$

We chose the IER distribution because it can model complex failure behaviors that simpler models, like the exponential or Rayleigh, cannot. Such models are limited to monotonic hazard rates and often fail to represent the nuanced life cycles of real components. The IER excels here, handling both decreasing and unimodal hazard functions. Its capacity to model a unimodal (or “upside-down bathtub”) hazard rate is particularly valuable, as this shape describes phenomena where the risk of failure first rises to a peak and then declines–a pattern seen in temporary stress events, like the operational load on a component during a specific mission.

**Fig. 1:**
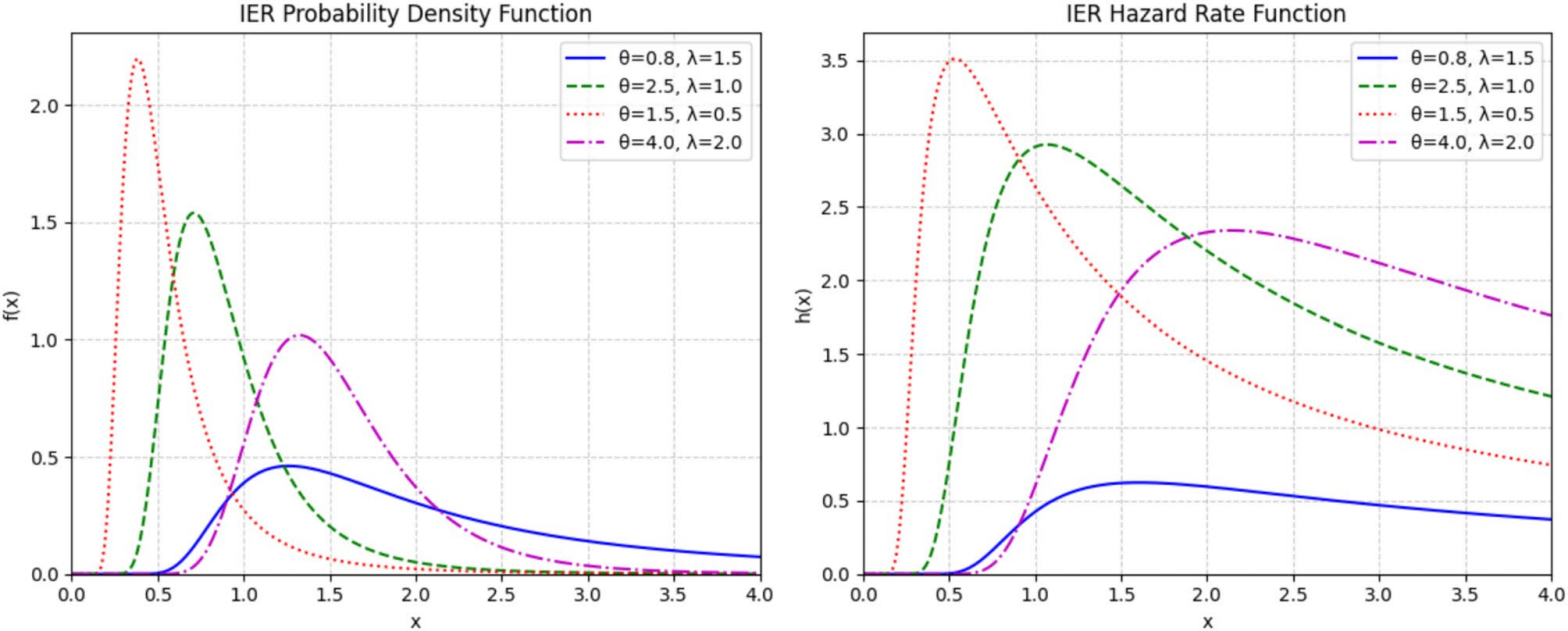
Visual illustration of the IER distribution’s flexibility. The left panel shows the PDF’s ability to model various forms of skewness, while the right panel demonstrates the HRF’s capacity to capture non-monotonic failure rates, including the unimodal (upside-down bathtub) shape that is a key feature motivating its use in this study.

Figure 1 visually demonstrates this versatility. The PDF can be adjusted to fit various forms of skewness, and the HRF confirms the unimodal hazard shape for different parameter settings. Because stress and strength often originate from distinct physical processes, the IER’s robustness allows us to characterize their diverse behaviors within a single, unified framework. A significant analytical advantage also comes from its closed-form CDF (Eq. (2.2)), which greatly simplifies the derivation of both the stress-strength reliability and the likelihood functions needed for complex data structures. This combination of practical flexibility and analytical tractability, which has been leveraged in other studies^11^, makes the IER distribution a theoretically sound and highly relevant foundation for our work.

### The multicomponent stress-strength model

The classic stress-strength model, defined as $\zeta = P(X > Y)$, is a foundational concept in reliability analysis^2^. However, modern systems are rarely single components; instead, they are complex assemblies of multiple parts that must all withstand operational stresses. This reality has motivated the extension of the classic model to a multicomponent stress-strength framework, which is now a central focus of reliability theory^4,3^.

In this study, we consider a coherent system made up of $\kappa$ independent and identically distributed (iid) strength components, $X_1, X_2, \dots , X_\kappa$, each subjected to a common stress, *Y*. We adopt the “*s*-out-of-$\kappa$:G” system configuration, a practical model for systems with built-in redundancy. Under this configuration, the system functions as long as at least *s* of its $\kappa$ components have a strength greater than the applied stress ($1 \le s \le \kappa$). Examples include multi-engine aircraft that can remain airborne with a minimum number of operational engines, or data storage arrays that maintain integrity despite several disk failures.

To formalize this, we assume the strength variables $\{X_i\}_{i=1}^\kappa$ are iid and follow an $\text {IER}(\theta _1, \lambda _1)$ distribution, while the stress variable *Y* follows an $\text {IER}(\theta _2, \lambda _2)$ distribution. All variables are assumed to be mutually independent. The reliability of this multicomponent system, which we denote by $\zeta _{s,\kappa }$, is the probability that at least *s* components have a strength exceeding the stress. If we let *N* be the number of components for which $X_i > Y$, then because the components are iid, the probability that any single component withstands the stress is a constant, $\vartheta = P(X > Y)$. This implies that *N* follows a binomial distribution with parameters $\kappa$ and $\vartheta$. The system reliability is therefore given by the binomial cumulative probability:

2.5 $$\begin{aligned} \zeta _{s,\kappa } = P(N \ge s) = \sum _{j=s}^{\kappa } \left( {\begin{array}{c}\kappa \\ j\end{array}}\right) \vartheta ^j (1-\vartheta )^{\kappa -j}. \end{aligned}$$

The core of this expression is the single-component reliability, $\vartheta$, which is found by integrating the survival function of *X* against the probability density function of *Y*:

2.6 $$\begin{aligned} \vartheta = \int _0^\infty S_X(y) f_Y(y) dy = \int _0^\infty \left( 1 - e^{-\lambda _1/y^2}\right) ^{\theta _1} \left[ 2\theta _2\lambda _2 y^{-3} e^{-\lambda _2/y^2} \left( 1 - e^{-\lambda _2/y^2}\right) ^{\theta _2-1} \right] dy. \end{aligned}$$

This integral typically does not have a closed-form solution. However, a closed-form expression can be obtained under the practical assumption that the scale parameters are equal, i.e., $\lambda _1 = \lambda _2 = \lambda$. This condition is often justified in experiments where stress and strength are measured on the same scale or are subject to similar environmental factors. With this assumption, the substitution $u = (1-e^{-\lambda /y^2})$ simplifies the integral to:

$$\vartheta = \frac{\theta _2}{\theta _1 + \theta _2}.$$

This result gives the probability of single-component success as $\vartheta = \frac{\theta _2}{\theta _1 + \theta _2}$, and the corresponding probability of failure as $1 - \vartheta = \frac{\theta _1}{\theta _1 + \theta _2}$. Substituting these probabilities into the binomial sum in Eq. (2.5) yields the final expression for the multicomponent reliability:

2.7 $$\begin{aligned} \zeta _{s,\kappa }= \sum _{j=s}^{\kappa } \left( {\begin{array}{c}\kappa \\ j\end{array}}\right) \frac{\theta _2^j \theta _1^{\kappa -j}}{(\theta _1 + \theta _2)^\kappa }. \end{aligned}$$

This function, which depends only on the shape parameters $\theta _1$ and $\theta _2$, is the central quantity of interest in our study. The subsequent sections are dedicated to developing frequentist and Bayesian methods for its estimation from the complex data structures unique to this research.

### Observed data structure

The theoretical models in reliability analysis are only as good as the data used to fit them. In practice, collecting complete lifetime data for every component is often infeasible due to high costs, long experimental durations, or the physical constraints of testing facilities. To address these challenges, modern reliability studies employ advanced data collection strategies, known as censoring and record-value schemes, to maximize information gain while maintaining experimental efficiency.

#### Strength data (X-sample): block adaptive type-II progressive hybrid censoring

For the strength component, we adopt a BAIIPH. This is a highly flexible, multi-stage approach that synthesizes several state-of-the-art censoring techniques to create a realistic and efficient testing protocol^26^. The BAIIPH scheme can be understood by breaking it down into its core components:

1. Block censoring: To manage facility limitations, the total of *N* units are divided into *g* smaller, independent groups (or “blocks”), where the *i*-th block contains $n_i$ units ($\sum _{i=1}^g n_i = N$). These blocks are tested in parallel, often in different facilities^26,19^.
2. Adaptive Type-II progressive hybrid censoring: Each block *i* is subjected to an adaptive hybrid censoring plan. This plan is designed to guarantee the observation of a predetermined number of failures, $s_i,$ while offering flexibility in the experimental duration^15^. A pre-specified progressive censoring plan $\textbf{R}_i = (R_{i,1}, R_{i,2}, \dots , R_{i,s_i})$ is set, where $R_{i,j}$ units are scheduled for removal at the time of the *j*-th failure, $x_{i,j:s_i}$. A maximum ideal test time, $T_i$, is also specified for each block. The “adaptive” procedure for each block *i* unfolds in one of two ways, as detailed in^26^:
  Case I: Experiment completes early. If the $s_i$-th failure occurs before the time limit $T_i$ (i.e., $x_{i,s_i:s_i} < T_i$), the experiment for that block is terminated. The censoring is carried out exactly as planned, and the remaining $R_{i,s_i} = n_i - s_i - \sum _{j=1}^{s_i-1} R_{i,j}$ units are removed.
  Case II: Experiment time is extended. If the $s_i$-th failure has not occurred by time $T_i$, let $J_i$ be the number of failures observed up to that point. To ensure the target of $s_i$ failures is met, the plan is adapted: the intermediate removals $R_{i,J_i+1}, \dots , R_{i,s_i-1}$ are cancelled (set to 0). The experiment continues past $T_i$ until the $s_i$-th failure is observed at time $x_{i,s_i:s_i}$. At this point, all remaining $R^*_{i,s_i} = n_i - s_i - \sum _{j=1}^{J_i} R_{i,j}$ units are removed.

* Likelihood function for strength data*: Given that the *g* blocks are independent, the total likelihood function for the strength data is the product of the likelihoods from each block. Let the PDF and survival function of the strength variable be $f_X(\cdot ; \theta _1, \lambda _1)$ and $S_X(\cdot ; \theta _1, \lambda _1)$, respectively. The data from block *i* is denoted by $\textbf{x} = (x_{i,1:s_i}, \dots , x_{i,s_i:s_i})$. The likelihood function for the entire strength dataset $\textbf{x}$ is:

2.8 $$\begin{aligned} \begin{aligned} L_X(\theta _1, \lambda _1 | \textbf{x}) = {}&\prod _{i=1}^{g} C_i \left( \prod _{j=1}^{s_i} f_X(x_{i,j}; \theta _1, \lambda _1) \right) \left( \prod _{j=1}^{J_i} \left( S_X(x_{i,j}; \theta _1, \lambda _1)\right) ^{R_{i,j}} \right) \\&\times \left( S_X(x_{i,s_i}; \theta _1, \lambda _1)\right) ^{R^*_{i,s_i}}, \end{aligned} \end{aligned}$$

where the censoring plan for block *i* is $\textbf{R}_i = (R_{i,1}, \dots , R_{i,s_i})$. The values of $R_{i,j}$ are determined by the adaptive scheme described above (i.e., some may be set to 0 in Case II) and $C_i=\prod _{j=1}^{s_i}\left( n_i -j+1-\sum _{l=1}^{\min (j-1,J_i)}R_{i,l} \right)$ is a normalization constant.

#### Stress data (Y-sample): upper k-records with inter-k-record times

For the stress variable, we use a data collection model based on upper k-records with inter-k-record times. Standard stress-strength analysis often assumes a random sample of stress values. However, in many applications, system failure is triggered by extreme stress events. Record value theory provides a natural framework for modeling such extremes^20^. A k-record is a value that ranks among the top *k* observations seen thus far. By collecting data as k-records, we focus on the most severe stresses a system is likely to encounter. Our model goes a step further by incorporating the inter-k-record times–the time intervals between the occurrences of these successive k-records. This temporal dimension provides richer information than the record values alone, as it helps characterize the frequency and clustering of extreme stress events, a concept that has proven valuable in reliability estimation^4,23^. The observed stress data, therefore, consists of a sequence of increasing record values. In this scheme, we observe a sequence of stress measurements. A value is identified as an upper k-record if it is among the *k* largest values observed to date. The term “inter-k-record times” refers to the number of observations (or time) between successive k-records. As shown by MirMostafaee et al.^27^, this additional temporal information can be used to significantly improve the precision of nonparametric or conditional inference. While we acknowledge this important feature, for the purpose of parametric estimation of the underlying stress distribution, the likelihood function is based on the sequence of the record values themselves.

*Likelihood function for stress data*: Let $U_{1},U_{2},\ldots ,U_{a}$ be iid continuous random variables from IER $(\theta _2, \lambda _2)$ distribution. The sequence of upper *k*-record values is defined as $Y_{i}=U_{T_{i}-k+1:T_{i}}$, $i=1,2,\ldots ,n$, $a>n$ where $T_{1}=k$ with probability 1, and for $i\ge 2$,

$T_{i}=\min \left\{ j:j> T_{i-1}, U_{j}> U_{T_{i-1}-k+1:T_{i-1}}\right\}$. For $k=1$, the k-records simply become the usual records. Moreover, the corresponding upper inter-k-record times are defined as $\delta _{i}=T_{i+1}-T_{i}$, $i\ge 1$. Let the set of upper k-records be denoted by $\textbf{y}= (y_{1},y_{2},\ldots ,y_{n})$ and the corresponding set of inter-k-record times by $\mathbf {\delta }= (\delta _{1},\delta _{2},\ldots ,\delta _{n-1})\in \mathbb {N}^{n-1}$. This structure is designed to capture information about extreme stress events. As established by Arnold et al.^20^, the joint likelihood function for the observed records and inter-record times, given that the underlying stress variable *Y* follows an $\text {IER}(\theta _2, \lambda _2)$ distribution, is:

2.9 $$\begin{aligned} L_Y(\theta _2, \lambda _2 | \textbf{y}, \mathbf {\delta }) = k^n \left( \prod _{i=1}^{n} \frac{f_Y(y_i; \theta _2, \lambda _2)}{S_Y(y_i; \theta _2, \lambda _2)} \left[ S_Y(y_i; \theta _2, \lambda _2)\right] ^k \right) \left( \prod _{i=1}^{n-1}\left[ 1-\left( S_Y(y_i; \theta _2, \lambda _2)\right) ^{k}\right] ^{\delta _{i}-1} \right) . \end{aligned}$$

This expression can be written more compactly in terms of the hazard rate function, $h_Y(y_i) = f_Y(y_i)/S_Y(y_i)$:

2.10 $$\begin{aligned} L_Y(\theta _2, \lambda _2 | \textbf{y}, \mathbf {\delta }) = k^n \left( \prod _{i=1}^{n} h_Y(y_i; \theta _2, \lambda _2) \left[ S_Y(y_i; \theta _2, \lambda _2)\right] ^k \right) \left( \prod _{i=1}^{n-1}\left[ 1-\left( S_Y(y_i; \theta _2, \lambda _2)\right) ^{k}\right] ^{\delta _{i}-1} \right) . \end{aligned}$$

Since the multicomponent failure strength and stress data are assumed to be collected independently, the total likelihood function for all parameters is the product of the individual likelihoods:

2.11 $$\begin{aligned} \begin{aligned} L(\theta _1, \theta _2, \lambda | \textbf{x},\textbf{y},\mathbf {\delta }) =&\quad \prod _{\tau =1}^{n} \prod _{i=1}^{g_{\tau }} \Biggl [ C_i \left( \prod _{j=1}^{s_i} f_X(x_{\tau , ij}; \theta _1, \lambda ) \right) \\&\qquad \times \left( \prod _{j=1}^{J_i} [S_X(x_{\tau , ij}; \theta _1, \lambda )]^{R_{i,j}} \right) [S_X(x_{\tau , is_i}; \theta _1, \lambda )]^{R^*_{i,s_i}} \Biggr ] \\&\qquad \times k^n \left( \prod _{i=1}^{n} h_Y(y_i; \theta _2, \lambda ) \left[ S_Y(y_i; \theta _2, \lambda )\right] ^k \right) \\&\qquad \times \left( \prod _{i=1}^{n-1}\left[ 1-\left( S_Y(y_i; \theta _2, \lambda )\right) ^{k}\right] ^{\delta _{i}-1} \right) . \end{aligned} \end{aligned}$$

**Scheme 1:**
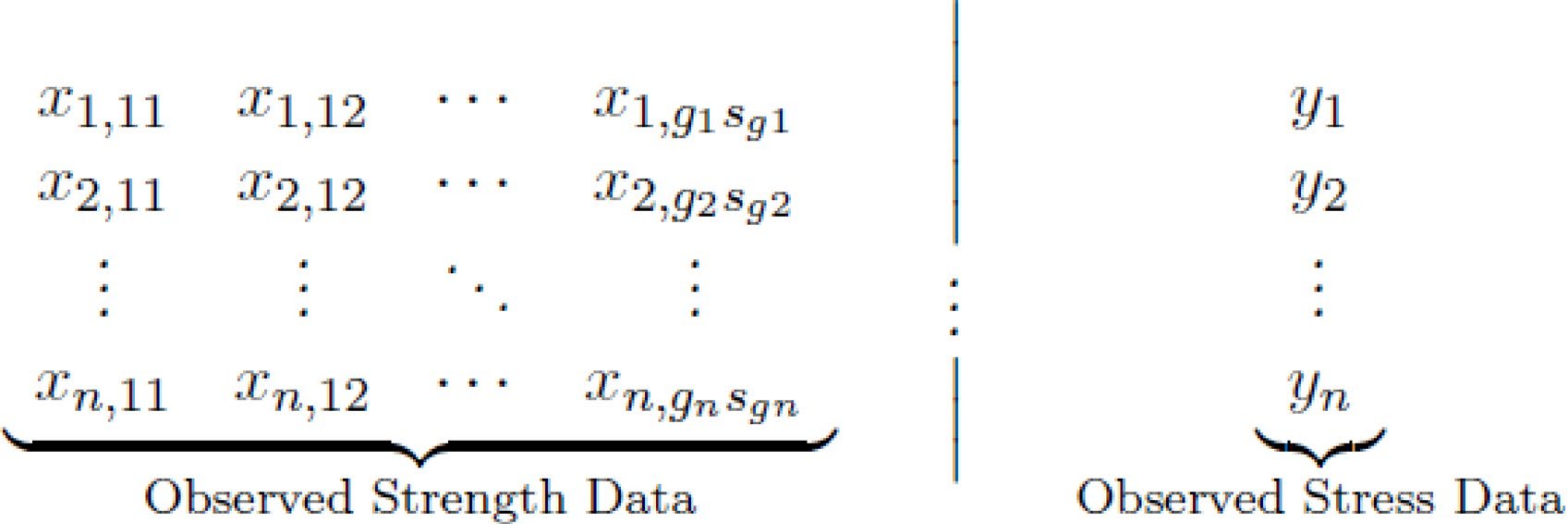
Observed multicomponent stress-strength data structure. Strength data are collected under BAIIPH censoring, while stress data are collected as URIT.

To summarize, the complete dataset for our analysis is composed of two distinct and independently gathered components, as illustrated in Scheme 1. The structure of the observed data is as follows:

- Strength data ($\textbf{x}$): The data on the left represent the failure times from *n* independent multicomponent life-testing experiments. For each experiment $\tau \in \{1, 2, \dots , n\}$, the observed data consists of the ordered failure times $\{x_{\tau , 11}, x_{\tau , 12}, \dots , x_{\tau , g_{\tau } s_{g\tau }}\}$ collected from $g_\tau$ blocks of an $s_{i}$-out-of-$\kappa$:G system under the BAIIPH censoring scheme.
- Stress data ($\textbf{y}, \mathbf {\delta }$): The data on the right represent the extreme stress values. This consists of a sequence of *n* upper *k*-record values, $\{y_1, y_2, \dots , y_n\}$, and their corresponding inter-k-record times.

The strength variables $\{x_{\tau ,ij}\}$ and stress variables $\{y_i\}$ are assumed to be independent. This comprehensive data structure provides a rich foundation for the inferential procedures developed in the remainder of this paper.

## Estimation of multicomponent stress-strength reliability

With the model’s theoretical components established, we turn to the primary goal of this study: statistical inference for the system reliability, $\zeta _{s,\kappa }$. This section develops a comprehensive set of estimation procedures from both frequentist and Bayesian viewpoints. We begin with the frequentist approach by deriving the MLE for $\zeta _{s,\kappa }$ and leveraging this result to construct asymptotic and bootstrap-based confidence intervals. We then transition to the Bayesian framework to derive posterior estimates for the parameters and the reliability function itself. This involves using Tierney and Kadane’s approximation for point estimation under different loss functions and a Metropolis-Hastings algorithm to generate samples for constructing credible intervals. Together, these methods provide a robust toolkit for assessing system reliability under the complex data conditions defined in this paper.

### Maximum likelihood estimation of $\zeta _{s,\kappa }$

Maximum likelihood estimators are foundational in frequentist inference due to their desirable asymptotic properties of consistency and efficiency. Our objective is to derive the MLE for the multicomponent stress-strength reliability, $\zeta _{s,\kappa }$, using the two independent datasets previously described: the strength data $\textbf{x}$ from the BAIIPH scheme^26^, and the stress data $(\textbf{y}, \boldsymbol{\delta })$ from the URIT scheme^20^. The model assumes that both strength (*X*) and stress (*Y*) variables follow IER distributions with a common scale parameter, such that $X \sim \text {IER}(\theta _1, \lambda )$ and $Y \sim \text {IER}(\theta _2, \lambda )$^24^.

Since the strength and stress data are collected independently, the total likelihood function for the parameter vector $\boldsymbol{\Theta } = (\theta _1, \theta _2, \lambda )$ is the product of the individual likelihoods. From Eq. (2.11), the combined likelihood function is:

3.1 $$\begin{aligned} L(\theta _1, \theta _2, \lambda \mid \text {data}) ={}&C_{\text {const}} \cdot \theta _1^{\sum _{\tau =1}^{n} \sum _{i=1}^{g_\tau } s_{\tau i}} \cdot \theta _2^n \cdot \lambda ^{n + \sum _{\tau =1}^{n} \sum _{i=1}^{g_\tau } s_{\tau i}} \nonumber \\&\times \exp \left( -\lambda \left\{ \sum _{\tau =1}^{n} \sum _{i=1}^{g_\tau } \sum _{j=1}^{s_{\tau i}} x_{\tau , ij}^{-2} + \sum _{i=1}^n y_i^{-2} \right\} \right) \nonumber \\&\times \prod _{\tau =1}^{n} \prod _{i=1}^{g_\tau } \left( \prod _{j=1}^{s_{\tau i}} \left( 1 - e^{-\lambda / x_{\tau , ij}^2} \right) ^{\theta _1 - 1} \cdot \prod _{j=1}^{J_{\tau i}} \left( 1 - e^{-\lambda / x_{\tau , ij}^2} \right) ^{\theta _1 R_{ i,j}} \right. \nonumber \\&\hspace{2cm} \left. \cdot \left( 1 - e^{-\lambda / x_{\tau , i s_{\tau i}}^2} \right) ^{\theta _1 R^*_{i ,s_{i}}} \right) \nonumber \\&\times \prod _{i=1}^{n} \left( 1 - e^{-\lambda / y_i^2} \right) ^{k\theta _2 - 1} \cdot \prod _{i=1}^{n-1} \left[ 1 - \left( 1 - e^{-\lambda / y_i^2} \right) ^{k\theta _2} \right] ^{\delta _i - 1}, \end{aligned}$$

where $$C_{\text {const}} = \left( \prod _{\tau =1}^{n} \prod _{i=1}^{g_\tau } C_i \right) \cdot k^n \cdot 2^{n+\sum _{\tau =1}^{n} \sum _{i=1}^{g_\tau } s_i} \cdot \left( \prod _{\tau =1}^{n} \prod _{i=1}^{g_\tau } \prod _{j=1}^{s_i} x_{\tau , ij}^{-3} \right) \cdot \left( \prod _{i=1}^n y_i^{-3} \right)$$.

For computational tractability, we work with the log-likelihood function, $\ell (\boldsymbol{\Theta }) = \log L(\boldsymbol{\Theta })$, which is given by:

3.2 $$\begin{aligned} \ell (\theta _1, \theta _2, \lambda |\, \text {data}) =&\ln (C_{\text {const}}) + \left( \sum _{\tau =1}^{n} \sum _{i=1}^{g_\tau } s_i\right) \ln (\theta _1) + n \ln (\theta _2) \nonumber \\&+ \left( n+\sum _{\tau =1}^{n} \sum _{i=1}^{g_\tau } s_i\right) \ln (\lambda ) - \lambda \left[ \sum _{\tau =1}^{n}\sum _{i=1}^{g_\tau }\sum _{j=1}^{s_i} x_{\tau , ij}^{-2} + \sum _{i=1}^n y_i^{-2} \right] \nonumber \\&+ \sum _{\tau =1}^{n} \sum _{i=1}^{g_\tau } \left[ \sum _{j=1}^{s_i} (\theta _1-1) \ln (1 - e^{-\lambda /x_{\tau , ij}^2}) + \sum _{j=1}^{J_i} \theta _1 R_{i,j} \ln (1 - e^{-\lambda /x_{\tau , ij}^2}) \right. \nonumber \\&\qquad \qquad \left. + \theta _1 R^*_{i,s_i} \ln (1 - e^{-\lambda /x_{\tau , is_i}^2}) \right] + \sum _{i=1}^{n} (k\theta _2 - 1) \ln (1 - e^{-\lambda /y_i^2}) \nonumber \\&+ \sum _{i=1}^{n-1} (\delta _{i}-1) \ln \left[ 1 - (1 - e^{-\lambda /y_i^2})^{k\theta _2} \right] . \end{aligned}$$

The MLEs of the parameters, denoted $\hat{\boldsymbol{\Theta }}=(\hat{\theta }_1, \hat{\theta }_2,\hat{\lambda })$, are the values that maximize this function. They are found by solving the system of non-linear equations obtained by setting the partial derivatives of $\ell$ to zero:

3.3 $$\begin{aligned} \frac{\partial \ell }{\partial \theta _1} = 0, \quad \frac{\partial \ell }{\partial \theta _2} = 0, \quad \frac{\partial \ell }{\partial \lambda } = 0. \end{aligned}$$

Letting $S_{\text {total}} = \sum _{\tau =1}^{n} \sum _{i=1}^{g_\tau } s_i$ be the total number of failures from the strength experiments, the resulting likelihood equations are as follows.

Differentiating $\ell$ with respect to $\theta _1$ gives:

$$\begin{aligned} \ell _{1}=\frac{\partial \ell }{\partial \theta _1} = \frac{S_{\text {total}}}{\theta _1}&+ \sum _{\tau =1}^{n} \sum _{i=1}^{g_\tau } \Biggl [ \sum _{j=1}^{s_i} \log (1 - e^{-\lambda /x_{\tau , ij}^2}) + \sum _{j=1}^{J_i} R_{i,j} \log (1 - e^{-\lambda /x_{\tau , ij}^2}) \\&\qquad + R^*_{i,s_i} \log (1 - e^{-\lambda /x{\tau , is_i}^2}) \Biggr ] = 0. \end{aligned}$$

Differentiating with respect to $\theta _2$ gives:

$$\begin{aligned} \ell _{2}=\frac{\partial \ell }{\partial \theta _2} = \frac{n}{\theta _2}&+ \sum _{i=1}^{n} k \log (1 - e^{-\lambda /y_i^2}) - \sum _{i=1}^{n-1} (\delta _{i}-1) \frac{k(1 - e^{-\lambda /y_i^2})^{k\theta _2} \log (1 - e^{-\lambda /y_i^2})}{1 - (1 - e^{-\lambda /y_i^2})^{k\theta _2}} = 0. \end{aligned}$$

Finally, defining $\Psi (z) = \frac{z^{-2}e^{-\lambda /z^2}}{1-e^{-\lambda /z^2}}$ for clarity, the derivative with respect to $\lambda$ is:

$$\begin{aligned} \ell _{3}=\frac{\partial \ell }{\partial \lambda } =&\frac{n+S_{\text {total}}}{\lambda } - \left[ \sum _{\tau =1}^{n}\sum _{i=1}^{g_\tau }\sum _{j=1}^{s_i} x_{\tau , ij}^{-2} + \sum _{i=1}^n y_i^{-2} \right] + \sum _{i=1}^{n} (k\theta _2 - 1) \Psi (y_i) \\&+ \sum _{\tau =1}^{n} \sum _{i=1}^{g_\tau } \left[ \sum _{j=1}^{s_i} (\theta _1-1) \Psi (x_{\tau , ij}) + \sum _{j=1}^{J_i} \theta _1 R_{i,j} \Psi (x_{\tau , ij}) + \theta _1 R^*_{i,s_i} \Psi (x_{\tau , is_i}) \right] \\&+ \sum _{i=1}^{n-1} (\delta _{i}-1) \frac{k\theta _2 (1 - e^{-\lambda /y_i^2})^{k\theta _2 - 1} (y_i^{-2} e^{-\lambda /y_i^2})}{1 - (1 - e^{-\lambda /y_i^2})^{k\theta _2}} = 0. \end{aligned}$$

Due to the highly non-linear and intricate form of these partial derivatives, an analytical solution for $\hat{\theta }_1, \hat{\theta }_2,$ and $\hat{\lambda }$ is not available. We must therefore employ iterative numerical optimization algorithms, such as the Newton-Raphson or quasi-Newton methods, to find the MLEs numerically.

Once the parameter estimates $\hat{\boldsymbol{\Theta }}$ are obtained, the MLE of the multicomponent reliability $\zeta _{s,\kappa }$ follows from the *invariance property* of MLEs. This property allows us to find the MLE of a function of parameters by simply evaluating the function at the parameter MLEs. Applying this to Eq. (2.7), the MLE of $\zeta _{s,\kappa }$ is:

3.4 $$\begin{aligned} \hat{\zeta }_{s,\kappa , \text {MLE}} = \sum _{j=s}^{\kappa } \left( {\begin{array}{c}\kappa \\ j\end{array}}\right) \frac{\hat{\theta }_2^j \hat{\theta }_1^{\kappa -j}}{(\hat{\theta }_1 + \hat{\theta }_2)^\kappa }. \end{aligned}$$

This point estimate, $\hat{\zeta }_{s,\kappa , \text {MLE}}$, is the foundation for constructing the confidence intervals discussed in the following sections and is consistent with methods used for other complex stress-strength models^4^.

Remark 1: Let the log-likelihood function be denoted by $\ell (\boldsymbol{\Theta } | \text {data})$, where $\boldsymbol{\Theta } = (\theta _1, \theta _2, \lambda )$. If a solution $\hat{\boldsymbol{\Theta }} = (\hat{\theta }_1, \hat{\theta }_2, \hat{\lambda })$ to the system of likelihood equations $\nabla \ell (\boldsymbol{\Theta }) = \textbf{0}$ exists within the parameter space $\Omega = \{ (\theta _1, \theta _2, \lambda ): \theta _1> 0, \theta _2> 0, \lambda > 0 \}$, then this solution is the unique maximum likelihood estimator of $\boldsymbol{\Theta }$. The uniqueness of this solution is established by demonstrating that the log-likelihood function $\ell (\boldsymbol{\Theta })$ is strictly concave at $\hat{\boldsymbol{\Theta }}$. This requires showing that its Hessian matrix, $\textbf{H}$, is negative definite when evaluated at $\hat{\boldsymbol{\Theta }}$. The Hessian matrix is given by:

$$\begin{aligned} \textbf{H} = \begin{pmatrix} \ell _{11} & \ell _{12} & \ell _{13} \\ \ell _{21} & \ell _{22} & \ell _{23} \\ \ell _{31} & \ell _{32} & \ell _{33} \end{pmatrix}, \end{aligned}$$

where $\ell _{ij} = \frac{\partial ^2 \ell }{\partial \theta _i \partial \theta _j}$, with $(\theta _1, \theta _2, \theta _3)$ corresponding to $(\theta _1, \theta _2, \lambda )$. For $\textbf{H}$ to be negative definite, its leading principal minors must satisfy the conditions $H_1 < 0$, $H_2 > 0$, and $H_3 < 0$. The relevant second-order partial derivatives, evaluated at $\hat{\boldsymbol{\Theta }}$, are as follows:

$$\begin{aligned} \ell _{11}&= \frac{\partial ^2 \ell }{\partial \theta _1^2} = -\frac{S_{\text {total}}}{\theta _1^2}, \\ \ell _{12}&= \frac{\partial ^2 \ell }{\partial \theta _1 \partial \theta _2} = 0, \\ \ell _{22}&= \frac{\partial ^2 \ell }{\partial \theta _2^2} = -\frac{n}{\theta _2^2} - k^2 \sum _{i=1}^{n-1} \frac{(\delta _i - 1)(1 - e^{-\lambda /y_i^2})^{k\theta _2} [\ln (1 - e^{-\lambda /y_i^2})]^2}{[1 - (1 - e^{-\lambda /y_i^2})^{k\theta _2}]^2}. \end{aligned}$$

The remaining derivatives are:

$$\begin{aligned} \ell _{13}&= \frac{\partial ^2 \ell }{\partial \theta _1 \partial \lambda } = \sum _{\tau =1}^{n} \sum _{i=1}^{g_\tau } \left[ \sum _{j=1}^{s_i} (1+R_{i,j}) \Psi (x_{\tau , ij}, \lambda ) + R^*_{i,s_i} \Psi (x_{\tau , is_i}, \lambda ) \right] , \\ \ell _{23}&= \frac{\partial ^2 \ell }{\partial \theta _2 \partial \lambda } = k \sum _{i=1}^{n} \Psi (y_i, \lambda ) \\&\quad \qquad \qquad + k \sum _{i=1}^{n-1} \frac{(\delta _i - 1) \Psi (y_i, \lambda )(1-e^{-\lambda /y_i^2})^{k\theta _2}\left[ 1+k\theta _2\ln (1-e^{-\lambda /y_i^2}) - (1-e^{-\lambda /y_i^2})^{k\theta _2}\right] }{\left[ 1-(1-e^{-\lambda /y_i^2})^{k\theta _2}\right] ^2},\\ \ell _{33}&= \frac{\partial ^2 \ell }{\partial \lambda ^2} = -\frac{n+S_{\text {total}}}{\lambda ^2}+ \sum _{i=1}^{n} (k\theta _2-1)\Psi '(y_i, \lambda ) \\&\quad + \sum _{\tau =1}^{n} \sum _{i=1}^{g_\tau } \left[ \sum _{j=1}^{s_i} (\theta _1(1+R_{i,j})-1) \Psi '(x_{\tau , ij}, \lambda ) + \theta _1 R^*_{i,s_i} \Psi '(x_{\tau , is_i}, \lambda ) \right] \\&\quad + \sum _{i=1}^{n-1} (\delta _i - 1) \frac{k\theta _2 (1-e^{-\lambda /y_i^2})^{k\theta _2}}{\left[ 1-(1-e^{-\lambda /y_i^2})^{k\theta _2}\right] ^2} \left[ k\theta _2 (\Psi (y_i, \lambda ))^2 + \Psi '(y_i, \lambda )\left( 1-(1-e^{-\lambda /y_i^2})^{k\theta _2}\right) \right] , \end{aligned}$$

where $\Psi (z, \lambda ) = \frac{z^{-2}e^{-\lambda /z^2}}{1-e^{-\lambda /z^2}}$ and its derivative $\Psi '(z, \lambda ) = \frac{-z^{-4}e^{-\lambda /z^2}}{(1-e^{-\lambda /z^2})^2}$ are always negative. Given these components, we can check the conditions for negative definiteness at the solution $\hat{\boldsymbol{\Theta }}$: The negative definiteness of $\textbf{H}$ is verified by inspecting its leading principal minors. The first minor, $H_1 = \ell _{11}$, is clearly negative. Since $\ell _{12}=0$, the second minor is $H_2 = \ell _{11}\ell _{22}$. Given that $\ell _{22}$ is also strictly negative (as a sum of negative terms), it follows that $H_2 > 0$. The third minor, $H_3 = \det (\textbf{H})$, simplifies to $H_3 = \ell _{11}\ell _{22}\ell _{33} - \ell _{11}\ell _{23}^2 - \ell _{22}\ell _{13}^2$. While its sign is not obvious from the algebraic form, it is determined by a fundamental property of likelihood theory. The observed information matrix, $\mathcal {I}(\boldsymbol{\Theta }) = -\textbf{H}$, is the sum of two positive definite matrices (from the independent strength and stress data) and is therefore itself positive definite. This requires its negative, the Hessian matrix $\textbf{H}$, to be negative definite, which in turn requires that $H_3 < 0$.

Since the principal minors alternate in sign as required ($H_1< 0, H_2 > 0, H_3 < 0$), the Hessian is negative definite. This confirms the concavity of the function and guarantees that the solution to the likelihood equations is a unique global maximum.

### Asymptotic confidence interval for $\zeta _{s,\kappa }$

Building upon the point estimation of the model parameters, we now develop an interval estimator for the multicomponent reliability $\zeta _{s,\kappa }$. Under certain regularity conditions, which our model satisfies, the MLEs are consistent and asymptotically normally distributed. Specifically, as the sample sizes increase, the distribution of the MLE vector $\hat{\boldsymbol{\Theta }} = (\hat{\theta }_1, \hat{\theta }_2, \hat{\lambda })^T$ approaches a multivariate normal distribution with a mean equal to the true parameter vector $\boldsymbol{\Theta }$ and a variance-covariance matrix given by the inverse of the Fisher information matrix, $\mathcal {I}(\boldsymbol{\Theta })^{-1}$^28^. While the expected Fisher information matrix can be difficult to compute, a standard and reliable alternative is to use the observed Fisher information matrix, which is estimated by the negative of the Hessian matrix evaluated at the MLEs, $\mathcal {I}(\hat{\boldsymbol{\Theta }}) = -\textbf{H}(\hat{\boldsymbol{\Theta }})$. The asymptotic variance-covariance matrix of the MLEs can therefore be estimated as:

3.5 $$\begin{aligned} \widehat{\text {Var}}(\hat{\boldsymbol{\Theta }}) = [-\textbf{H}(\hat{\boldsymbol{\Theta }})]^{-1} = \begin{pmatrix} \hat{\sigma }_{11} & \hat{\sigma }_{12} & \hat{\sigma }_{13} \\ \hat{\sigma }_{21} & \hat{\sigma }_{22} & \hat{\sigma }_{23} \\ \hat{\sigma }_{31} & \hat{\sigma }_{32} & \hat{\sigma }_{33} \end{pmatrix}, \end{aligned}$$

where $\hat{\sigma }_{ij}$ is the estimated asymptotic covariance between $\hat{\theta }_i$ and $\hat{\theta }_j$.

To find the asymptotic variance of our reliability estimator, $\hat{\zeta }_{s,\kappa }$, we employ the delta method. This technique approximates the variance of a function of random variables using a first-order Taylor series expansion. The asymptotic variance of $\hat{\zeta }_{s,\kappa }$ is given by:

3.6 $$\begin{aligned} \hat{\sigma }^2_{\hat{\zeta }_{s,\kappa }} \approx (\nabla \zeta _{s,\kappa })^T \widehat{\text {Var}}(\hat{\boldsymbol{\Theta }}) (\nabla \zeta _{s,\kappa }), \end{aligned}$$

where $\nabla \zeta _{s,\kappa }$ is the gradient of the reliability function evaluated at the MLEs. Since $\zeta _{s,\kappa }$ depends only on $\theta _1$ and $\theta _2$, the gradient vector is $\nabla \zeta _{s,\kappa } = (\frac{\partial \zeta _{s,\kappa }}{\partial \theta _1}, \frac{\partial \zeta _{s,\kappa }}{\partial \theta _2}, 0)^T$. The partial derivatives are:

3.7 $$\begin{aligned} \frac{\partial \zeta _{s,\kappa }}{\partial \theta _1}&= \sum _{j=s}^{\kappa } \left( {\begin{array}{c}\kappa \\ j\end{array}}\right) \frac{(\kappa -j)\theta _2^{j+1}\theta _1^{\kappa -j-1} - j\theta _2^j\theta _1^{\kappa -j}}{(\theta _1+\theta _2)^{\kappa +1}} ,\end{aligned}$$

3.8 $$\begin{aligned} \frac{\partial \zeta _{s,\kappa }}{\partial \theta _2}&= \sum _{j=s}^{\kappa } \left( {\begin{array}{c}\kappa \\ j\end{array}}\right) \frac{j\theta _2^{j-1}\theta _1^{\kappa -j+1} - (\kappa -j)\theta _2^j\theta _1^{\kappa -j}}{(\theta _1+\theta _2)^{\kappa +1}}. \end{aligned}$$

Let $d_1 = \frac{\partial \zeta _{s,\kappa }}{\partial \theta _1}\Big |_{\hat{\boldsymbol{\Theta }}}$ and $d_2 = \frac{\partial \zeta _{s,\kappa }}{\partial \theta _2}\Big |_{\hat{\boldsymbol{\Theta }}}$. The variance of $\hat{\zeta }_{s,\kappa }$ is then estimated by:

3.9 $$\begin{aligned} \hat{\sigma }^2_{\hat{\zeta }_{s,\kappa }} = d_1^2 \hat{\sigma }_{11} + d_2^2 \hat{\sigma }_{22} + 2d_1 d_2 \hat{\sigma }_{12}. \end{aligned}$$

A straightforward $100(1-\alpha )\%$ ACI for $\zeta _{s,\kappa }$ is then constructed as:

3.10 $$\begin{aligned} \hat{\zeta }_{s,\kappa } \pm z_{\alpha /2} \sqrt{\hat{\sigma }^2_{\hat{\zeta }_{s,\kappa }}}, \end{aligned}$$

where $z_{\alpha /2}$ is the upper $(\alpha /2)$-th percentile of the standard normal distribution. However, this interval may produce bounds outside the valid range of [0, 1]. To address this, we apply a logarithmic transformation, a technique used by^29^ and others, to ensure the interval remains within the proper bounds. Let $h(\zeta ) = \log (\frac{\zeta }{1-\zeta })$. The variance of $h(\hat{\zeta }_{s,\kappa })$ is found via the delta method:

3.11 $$\begin{aligned} \hat{\sigma }^2_{h(\hat{\zeta }_{s,\kappa })} \approx [h'(\hat{\zeta }_{s,\kappa })]^2 \hat{\sigma }^2_{\hat{\zeta }_{s,\kappa }} = \frac{\hat{\sigma }^2_{\hat{\zeta }_{s,\kappa }}}{[\hat{\zeta }_{s,\kappa }(1-\hat{\zeta }_{s,\kappa })]^2}. \end{aligned}$$

The $100(1-\alpha )\%$ ACI for the transformed parameter $h(\zeta _{s,\kappa })$ is:

3.12 $$\begin{aligned} \left( \log \left( \frac{\hat{\zeta }_{s,\kappa }}{1-\hat{\zeta }_{s,\kappa }}\right) - z_{\alpha /2} \frac{\sqrt{\hat{\sigma }^2_{\hat{\zeta }_{s,\kappa }}}}{\hat{\zeta }_{s,\kappa }(1-\hat{\zeta }_{s,\kappa })}, \quad \log \left( \frac{\hat{\zeta }_{s,\kappa }}{1-\hat{\zeta }_{s,\kappa }}\right) + z_{\alpha /2} \frac{\sqrt{\hat{\sigma }^2_{\hat{\zeta }_{s,\kappa }}}}{\hat{\zeta }_{s,\kappa }(1-\hat{\zeta }_{s,\kappa })} \right) . \end{aligned}$$

Let the lower and upper bounds of this interval be (*L*, *U*). Applying the inverse transformation (the logistic function), we obtain the final, more reliable $100(1-\alpha )\%$ ACI for $\zeta _{s,k}$: $\left( \frac{e^L}{1+e^L}, \; \frac{e^U}{1+e^U}\right)$.

### Bootstrap confidence intervals for $\zeta _{s,\kappa }$

While the asymptotic confidence interval developed in previous section offers a computationally straightforward approach, its validity hinges on the central limit theorem, which presumes that the sampling distribution of the MLEs is approximately normal. This assumption may not hold reliably for moderate sample sizes, especially given the intricate nature of the data collected under BAIIPH and k-record schemes. Such complex data structures can lead to skewed sampling distributions for $\hat{\zeta }_{s,\kappa }$. To construct more robust and accurate confidence intervals that do not depend on large-sample normality, we turn to resampling techniques, specifically parametric bootstrap methods^30^.

#### Percentile bootstrap (Boot-p) confidence interval for $\zeta _{s,\kappa }$

Due to the specific generative nature of our data collection schemes–BAIIPH for strength and URIT for stress–a standard non-parametric bootstrap (i.e., resampling observations with replacement) is unsuitable. Instead, a parametric bootstrap approach is required, as it simulates new datasets directly from the fitted model, thereby respecting the underlying data generation mechanisms. The procedure is outlined as follows:

1. *Initial estimation:* From the original observed data (strength data $\textbf{x}$ and stress data $\textbf{y}$ with inter-record times $\boldsymbol{\delta }$), compute the MLEs of the parameters, $\hat{\Theta } = (\hat{\theta }_1, \hat{\theta }_2, \hat{\lambda })$. Then, calculate the MLE of the system reliability, $\hat{\zeta }_{s,\kappa ,MLE}$, using Eq. (3.4).
2. *Bootstrap strength sample generation:* Generate a bootstrap strength sample, $\textbf{x}^*$, by simulating data from the IER($\hat{\theta }_1, \hat{\lambda }$) distribution. This simulation must precisely replicate the original BAIIPH scheme, including the number of groups ($g_\tau$), target failures ($s_i$), predetermined test times ($T_i$), and censoring plans ($R_{i,j}$), to produce a valid bootstrap censored sample $\textbf{x}^*$.
3. *Bootstrap stress sample generation:* Generate a bootstrap stress sample, $\textbf{y}^*$, by simulating data from the IER($\hat{\theta }_2, \hat{\lambda }$) distribution under the URIT scheme. This yields a new set of *n* upper k-records, $\textbf{y}^*$, and their corresponding inter-k-record times, $\boldsymbol{\delta }^*$.
4. *Bootstrap parameter estimation:* Using the generated bootstrap samples $\textbf{x}^*$ and $(\textbf{y}^*, \boldsymbol{\delta }^*)$, compute the bootstrap MLEs of the parameters, denoted as $\hat{\Theta }^* = (\hat{\theta }_1^*, \hat{\theta }_2^*, \hat{\lambda }^*)$.
5. *Bootstrap reliability calculation:* Calculate the bootstrap estimate of reliability, $\hat{\zeta }_{s,\kappa }^*$, by substituting the bootstrap MLEs ($\hat{\Theta }^*$) into the reliability function given by Eq. (3.4).
6. *Repetition:* Repeat Steps 2 through 5 a large number of times, *B* (e.g., $B=2000$), to obtain a collection of bootstrap reliability estimates: $\{\hat{\zeta }_{s,\kappa ,1}^*, \hat{\zeta }_{s,\kappa ,2}^*, \dots , \hat{\zeta }_{s,\kappa ,B}^*\}$.

This collection of *B* estimates serves as an empirical approximation of the sampling distribution of $\hat{\zeta }_{s,\kappa }$. The percentile bootstrap (Boot-p) interval is constructed directly from the quantiles of this empirical distribution^31^. Let $\hat{\zeta }_{s,\kappa ,(\alpha )}^*$ be the $100\alpha$-th percentile of the ordered bootstrap estimates. The $100(1-\alpha )\%$ Boot-p confidence interval for $\zeta _{s,\kappa }$ is given by:

3.13 $$\begin{aligned} \left( \hat{\zeta }_{s,\kappa ,(\alpha /2)}^*, \hat{\zeta }_{s,\kappa ,(1-\alpha /2)}^* \right) . \end{aligned}$$

This interval is simple to compute and performs well, particularly when the sampling distribution of $\hat{\zeta }_{s,\kappa }$ is symmetric and its estimator is median-unbiased.

#### Bootstrap-t (Boot-t) confidence interval for $\zeta _{s,\kappa }$

For potentially improved coverage accuracy, particularly in skewed distributions, the bootstrap-t (or studentized bootstrap) interval is an excellent alternative. It is known to be second-order accurate, a significant improvement over the first-order accuracy of the percentile and asymptotic intervals^32^. This method works by bootstrapping a studentized pivot quantity, which often has a more stable sampling distribution than the estimator itself. The process requires an estimate of the standard error for each bootstrap replicate:

1. *Studentized statistic calculation:* First, generate the collection of *B* bootstrap reliability estimates, $\{\hat{\zeta }_{s,\kappa ,1}^*, \dots , \hat{\zeta }_{s,\kappa ,B}^*\}$, as described in the Boot-p procedure. For each bootstrap sample $b=1, \dots , B$, compute the studentized statistic:
  3.14 $$\begin{aligned} T_b^* = \frac{\hat{\zeta }_{s,\kappa ,b}^* - \hat{\zeta }_{s,\kappa ,MLE}}{\widehat{se}(\hat{\zeta }_{s,\kappa ,b}^*)}. \end{aligned}$$
  Here, $\hat{\zeta }_{s,\kappa ,MLE}$ is the original MLE of the reliability. The term $\widehat{se}(\hat{\zeta }_{s,\kappa ,b}^*)$ is the standard error of the reliability estimate computed from the *b*-th bootstrap sample. This is obtained by applying the delta method (as detailed in previous section) to the bootstrap data and the bootstrap parameter estimates $\hat{\Theta }_b^*$.
2. Empirical distribution of T*: Obtain the empirical distribution of the studentized statistics from the *B* bootstrap samples: $\{T_1^*, T_2^*, \dots , T_B^*\}$.
3. *Quantile determination:* Find the $(\alpha /2)$ and $(1-\alpha /2)$ percentiles from the empirical distribution of $T^*$. Let these be denoted as $t_{\alpha /2}^*$ and $t_{1-\alpha /2}^*$, respectively.
4. *Interval construction:* The $100(1-\alpha )\%$ bootstrap-t confidence interval for $\zeta _{s,k}$ is then constructed as:
  3.15 $$\begin{aligned} \left( \hat{\zeta }_{s,\kappa ,MLE} - t_{1-\alpha /2}^* \widehat{se}(\hat{\zeta }_{s,\kappa ,MLE}), \quad \hat{\zeta }_{s,\kappa ,MLE} - t_{\alpha /2}^* \widehat{se}(\hat{\zeta }_{s,\kappa ,MLE}) \right) , \end{aligned}$$
  where $\widehat{se}(\hat{\zeta }_{s,\kappa ,MLE})$ is the standard error of the original MLE, calculated from the observed-information matrix as the square root of the variance given in Eq. (3.9).

### Bayes estimates for $\zeta _{s,\kappa }$

In contrast to the frequentist approach, which treats model parameters as fixed, unknown constants, the Bayesian paradigm considers them to be random variables. This allows us to incorporate prior knowledge about the parameters and update our beliefs in light of the observed data. The synthesis of prior information and data-driven evidence is encapsulated in the posterior distribution, which forms the basis for all subsequent inference.

To develop the Bayesian framework for our model, we must first specify prior distributions for the unknown parameters $\theta _1$, $\theta _2$, and $\lambda$. Since these parameters are positive, the gamma distribution is a natural and flexible choice for modeling our prior beliefs. We assume that $\theta _1$, $\theta _2$, and $\lambda$ are mutually independent and follow gamma distributions, defined as:

- $$\pi (\theta _1) \propto \theta _1^{a_1-1} e^{-b_1 \theta _1}$$, for $$\theta _1 > 0,$$
- $$\pi (\theta _2) \propto \theta _2^{a_2-1} e^{-b_2 \theta _2}$$, for $$\theta _2 > 0,$$
- $$\pi (\lambda ) \propto \lambda ^{a_3-1} e^{-b_3 \lambda }$$, for $$\lambda > 0.$$

The hyperparameters $(a_1, b_1, a_2, b_2, a_3, b_3)$ are chosen to reflect existing knowledge from previous studies, expert opinion, or physical constraints. In cases where little prior information is available, one can specify non-informative priors by setting the hyperparameters to values close to zero (e.g., $a_i, b_i \rightarrow 0$), which allows the likelihood function to dominate the posterior inference^33^. The joint posterior distribution is obtained by applying Bayes’ theorem, which states that the posterior is proportional to the product of the likelihood function and the joint prior distribution. Substituting the likelihood function from Eq. (3.1) and the gamma priors, we derive the joint posterior density:

3.16 $$\begin{aligned} \pi (\theta _1, \theta _2, \lambda \,|\, \text {data})&\propto \theta _1^{S_{\text {total}} + a_1 - 1} \theta _2^{n + a_2 - 1} \lambda ^{n + S_{\text {total}} + a_3 - 1} e^{-b_1 \theta _1 - b_2 \theta _2 - b_3 \lambda } e^{-\lambda \mathcal {W}} \nonumber \\&\quad \times \prod _{\tau =1}^n \prod _{i=1}^{g_\tau } \left[ \prod _{j=1}^{s_{\tau i}} \left( 1 - e^{-\lambda /x_{\tau ,ij}^2}\right) ^{\theta _1 - 1} \prod _{j=1}^{J_{\tau i}} \left( 1 - e^{-\lambda /x_{\tau ,ij}^2}\right) ^{\theta _1 R_{i,j}} \right. \nonumber \\&\qquad \qquad \left. \times \left( 1 - e^{-\lambda /x_{\tau ,is_{\tau i}}^2}\right) ^{\theta _1 R^*_{i,s_{ i}}} \right] \nonumber \\&\quad \times \prod _{i=1}^n \left( 1 - e^{-\lambda /y_i^2}\right) ^{k\theta _2 - 1} \prod _{i=1}^{n-1} \left[ 1 - \left( 1 - e^{-\lambda /y_i^2}\right) ^{k\theta _2} \right] ^{\delta _i - 1}, \end{aligned}$$

where $\mathcal {W} = \sum _{\tau =1}^n \sum _{i=1}^{g_\tau } \sum _{j=1}^{s_{\tau i}} x_{\tau ,ij}^{-2} + \sum _{i=1}^n y_i^{-2}$. The complexity of this posterior distribution makes it immediately clear that analytical integration is not feasible. To obtain a single point estimate for the reliability function $\zeta _{s,\kappa }$, we must evaluate the posterior distribution under a chosen loss function. The Bayes estimate is the value that minimizes the expected posterior loss.

Squared error loss function: The most common choice is the symmetric squared error loss function, $L(\zeta , \hat{\zeta }) = (\zeta - \hat{\zeta })^2$. Let $\boldsymbol{\Theta } = (\theta _1, \theta _2, \lambda )$ be the vector of parameters, and let $\Omega = (0, \infty )^3$ be the parameter space. The Bayes estimate under SE is then the posterior mean:

3.17 $$\begin{aligned} \hat{\zeta }_{s,\kappa ,SE} = E_{\pi }[\zeta _{s,\kappa } \,|\, \text {data}] = \int _{\Omega } \zeta _{s,\kappa }(\boldsymbol{\Theta }) \pi (\boldsymbol{\Theta } \,|\, \text {data}) \,d\boldsymbol{\Theta }. \end{aligned}$$

Linear exponential loss function: In reliability analysis, underestimation of reliability is often less critical than overestimation. The asymmetric LINEX loss function, $L(\zeta , \hat{\zeta }) = e^{c(\hat{\zeta } - \zeta )} - c(\hat{\zeta } - \zeta ) - 1$ for $c \ne 0$, accounts for this by assigning different penalties. The sign and magnitude of the shape parameter *c* control the direction and degree of asymmetry. For $c > 0$, overestimation is penalized more heavily. The Bayes estimate under LINEX loss is given by^34^:

3.18 $$\begin{aligned} \hat{\zeta }_{s,\kappa ,L} = -\frac{1}{c} \ln \left( E_{\pi }[e^{-c\zeta _{s,\kappa }} \,|\, \text {data}] \right) , \end{aligned}$$

where the posterior expectation is calculated as:

3.19 $$\begin{aligned} E_{\pi }[e^{-c\zeta _{s,\kappa }} \,|\, \text {data}] = \int _{\Omega } e^{-c\zeta _{s,\kappa }(\boldsymbol{\Theta })} \pi (\boldsymbol{\Theta } \,|\, \text {data}) \,d\boldsymbol{\Theta }. \end{aligned}$$

As is evident from the intricate form of the posterior distribution in Eq. (3.16), the three-dimensional integrals required to compute these Bayes estimates are analytically intractable. Therefore, we must rely on advanced numerical approximation techniques. The following subsections will detail two such methods: the Tierney and Kadane approximation for analytical tractability and a Metropolis-Hastings algorithm for posterior sampling.

#### Tierney and Kadane’s approximation for point estimates

As a powerful, deterministic alternative to computationally intensive simulation, we employ the Tierney and Kadane approximation^35^. This method is exceptionally effective for calculating the ratio of two integrals–the precise form of a posterior expectation. The core idea is to approximate the integrands with a Gaussian function, yielding a highly accurate result with minimal computational overhead.

The general TK framework: The posterior expectation of a function $U(\boldsymbol{\Theta })$ is given by the TK formula:

3.20 $$\begin{aligned} \hat{E}[U(\boldsymbol{\Theta })] = \sqrt{\frac{\det (\boldsymbol{\Sigma }^*)}{\det (\boldsymbol{\Sigma })}} \exp \left\{ \mathcal {L}^*(\boldsymbol{\Theta }^*) - \mathcal {L}(\hat{\boldsymbol{\Theta }}) \right\} , \end{aligned}$$

where $\mathcal {L}(\boldsymbol{\Theta }) = \ln (\pi (\boldsymbol{\Theta } \,|\, \text {data}))$ is the log-posterior, and the auxiliary function is $\mathcal {L}^*(\boldsymbol{\Theta }) = \mathcal {L}(\boldsymbol{\Theta }) + \ln (U(\boldsymbol{\Theta }))$. Implementation requires finding the modes ($\hat{\boldsymbol{\Theta }}$, $\boldsymbol{\Theta }^*$) and negative inverse Hessian ($\boldsymbol{\Sigma }$, $\boldsymbol{\Sigma }^*$) of these functions. This, in turn, requires their first and second derivatives.

Derivatives of the log-posterior: The log-posterior function, $\mathcal {L}(\boldsymbol{\Theta })$, is the sum of the log-likelihood $\ell (\boldsymbol{\Theta })$ from Eq. (3.2) and the log-priors. We denote the first partial derivatives of the log-likelihood as $\ell _i = \partial \ell / \partial \theta _i$ (where $\boldsymbol{\theta } = (\theta _1, \theta _2, \lambda )$) and the second partials as $\ell _{ij} = \partial ^2 \ell / \partial \theta _i \partial \theta _j$. The derivatives of the log-posterior are:

3.21 $$\begin{aligned} \frac{\partial \mathcal {L}}{\partial \theta _1}&= \ell _{1} + \frac{a_1 - 1}{\theta _1} - b_1,&\mathcal {L}_{11}&= \ell _{11} - \frac{a_1 - 1}{\theta _1^2}, \end{aligned}$$

3.22 $$\begin{aligned} \frac{\partial \mathcal {L}}{\partial \theta _2}&= \ell _{2} + \frac{a_2 - 1}{\theta _2} - b_2,&\mathcal {L}_{22}&= \ell _{22} - \frac{a_2 - 1}{\theta _2^2}, \end{aligned}$$

3.23 $$\begin{aligned} \frac{\partial \mathcal {L}}{\partial \lambda }&= \ell _{3} + \frac{a_3 - 1}{\lambda } - b_3,&\mathcal {L}_{33}&= \ell _{33} - \frac{a_3 - 1}{\lambda ^2}. \end{aligned}$$

Since the priors are independent, the mixed partial derivatives are unchanged: $\mathcal {L}_{ij} = \ell _{ij}$ for $i \ne j$.

Applying TK for SE loss: For the symmetric SE loss, the goal is to estimate the posterior mean by setting $U(\boldsymbol{\Theta }) = \zeta _{s,\kappa }(\theta _1, \theta _2)$. The auxiliary function to maximize is:

3.24 $$\begin{aligned} \mathcal {L}_{\text {SE}}^*(\boldsymbol{\Theta }) = \mathcal {L}(\boldsymbol{\Theta }) + \ln (\zeta _{s,\kappa }(\theta _1, \theta _2)). \end{aligned}$$

Let $\zeta _{s,\kappa }$ be denoted simply as $\zeta$, and let its partial derivatives be $\zeta _i = \partial \zeta /\partial \theta _i$ and $\zeta _{ij} = \partial ^2\zeta /\partial \theta _i\partial \theta _j$. The first partial derivatives of $\mathcal {L}_{\text {SE}}^*$ are:

3.25 $$\begin{aligned} \frac{\partial \mathcal {L}_{\text {SE}}^*}{\partial \theta _1}&= \frac{\partial \mathcal {L}}{\partial \theta _1} + \frac{\zeta _1}{\zeta }, \end{aligned}$$

3.26 $$\begin{aligned} \frac{\partial \mathcal {L}_{\text {SE}}^*}{\partial \theta _2}&= \frac{\partial \mathcal {L}}{\partial \theta _2} + \frac{\zeta _2}{\zeta }, \end{aligned}$$

3.27 $$\begin{aligned} \frac{\partial \mathcal {L}_{\text {SE}}^*}{\partial \lambda }&= \frac{\partial \mathcal {L}}{\partial \lambda }. \end{aligned}$$

The elements of the Hessian matrix for $\mathcal {L}_{\text {SE}}^*$ are:

3.28 $$\begin{aligned} \mathcal {L}_{\text {SE},11}^*&= \mathcal {L}_{11} + \frac{\zeta _{11}\zeta - \zeta _1^2}{\zeta ^2},&\mathcal {L}_{\text {SE},12}^*&= \mathcal {L}_{12} + \frac{\zeta _{12}\zeta - \zeta _1\zeta _2}{\zeta ^2}, \end{aligned}$$

3.29 $$\begin{aligned} \mathcal {L}_{\text {SE},22}^*&= \mathcal {L}_{22} + \frac{\zeta _{22}\zeta - \zeta _2^2}{\zeta ^2},&\mathcal {L}_{\text {SE},13}^*&= \mathcal {L}_{13}, \end{aligned}$$

3.30 $$\begin{aligned} \mathcal {L}_{\text {SE},33}^*&= \mathcal {L}_{33},&\mathcal {L}_{\text {SE},23}^*&= \mathcal {L}_{23}. \end{aligned}$$

With these derivatives, we can numerically find the mode $\boldsymbol{\Theta }_{\text {SE}}^*$ and the Hessian $\boldsymbol{\Sigma }_{\text {SE}}^*$ needed for the TK formula. The TK estimate for the reliability is then:

3.31 $$\begin{aligned} \hat{\zeta }_{s,k,TK-SE} = \sqrt{\frac{\det (\boldsymbol{\Sigma }_{SE}^*)}{\det (\boldsymbol{\Sigma })}} \exp \left\{ \mathcal {L}_{SE}^*(\boldsymbol{\Theta }_{SE}^*) - \mathcal {L}(\hat{\boldsymbol{\Theta }}) \right\} . \end{aligned}$$

Applying TK for LINEX loss: In reliability analysis, the cost of overestimating system performance (leading to unexpected failures) is often much higher than the cost of underestimating it (leading to conservative designs). The asymmetric LINEX loss function is ideal for such scenarios. The parameter *c* controls the direction and degree of asymmetry: a positive *c* penalizes overestimation more severely, while a negative *c* penalizes underestimation more. As $c \rightarrow 0$, LINEX loss converges to the symmetric SE loss.

For LINEX loss, we need to estimate $E[e^{-c\zeta _{s,\kappa }}]$, so we set $U(\boldsymbol{\Theta }) = e^{-c\zeta _{s,\kappa }(\theta _1, \theta _2)}$. This yields a simpler auxiliary function:

3.32 $$\begin{aligned} \mathcal {L}_{\text {L}}^*(\boldsymbol{\Theta }) = \mathcal {L}(\boldsymbol{\Theta }) - c\zeta _{s,\kappa }(\theta _1, \theta _2). \end{aligned}$$

The first partial derivatives of $\mathcal {L}_{\text {L}}^*$ are:

3.33 $$\begin{aligned} \frac{\partial \mathcal {L}_{\text {L}}^*}{\partial \theta _1}&= \frac{\partial \mathcal {L}}{\partial \theta _1} - c\zeta _1, \end{aligned}$$

3.34 $$\begin{aligned} \frac{\partial \mathcal {L}_{\text {L}}^*}{\partial \theta _2}&= \frac{\partial \mathcal {L}}{\partial \theta _2} - c\zeta _2, \end{aligned}$$

3.35 $$\begin{aligned} \frac{\partial \mathcal {L}_{\text {L}}^*}{\partial \lambda }&= \frac{\partial \mathcal {L}}{\partial \lambda }. \end{aligned}$$

The Hessian elements for $\mathcal {L}_{\text {L}}^*$ are also straightforward:

3.36 $$\begin{aligned} \mathcal {L}_{\text {L},11}^*&= \mathcal {L}_{11} - c\zeta _{11},&\mathcal {L}_{\text {L},12}^*&= \mathcal {L}_{12} - c\zeta _{12}, \end{aligned}$$

3.37 $$\begin{aligned} \mathcal {L}_{\text {L},22}^*&= \mathcal {L}_{22} - c\zeta _{22},&\mathcal {L}_{\text {L},13}^*&= \mathcal {L}_{13}, \end{aligned}$$

3.38 $$\begin{aligned} \mathcal {L}_{\text {L},33}^*&= \mathcal {L}_{33},&\mathcal {L}_{\text {L},23}^*&= \mathcal {L}_{23}. \end{aligned}$$

After finding the mode $\boldsymbol{\Theta }_{\text {L}}^*$ and Hessian $\boldsymbol{\Sigma }_{\text {L}}^*$ using these derivatives, we compute $\hat{E}[e^{-c\zeta _{s,\kappa }}]$ via Eq. (3.20). The final LINEX estimate is then:

3.39 $$\begin{aligned} \hat{\zeta }_{s,\kappa ,\text {TK-L}} = -\frac{1}{c} \ln \left( \sqrt{\frac{\det (\boldsymbol{\Sigma }_{\text {L}}^*)}{\det (\boldsymbol{\Sigma })}} \exp \left\{ \mathcal {L}_{\text {L}}^*(\boldsymbol{\Theta }_{\text {L}}^*) - \mathcal {L}(\hat{\boldsymbol{\Theta }}) \right\} \right) . \end{aligned}$$

While the Tierney and Kadane approximation^35^ offers an efficient way to derive point estimates, it cannot fully characterize the posterior uncertainty needed for interval estimation. To achieve a more complete picture of the posterior distributions for the model parameters and the reliability function $\zeta _{s,\kappa }$, we turn to simulation. The Metropolis-Hastings algorithm, a widely-used Markov Chain Monte Carlo (MCMC) method, is particularly well-suited for this problem, given the analytical intractability of the posterior distribution shown in Eq. (3.16)^33^.

#### MCMC algorithm for $\zeta _{s,\kappa }$

**Fig. 2:**
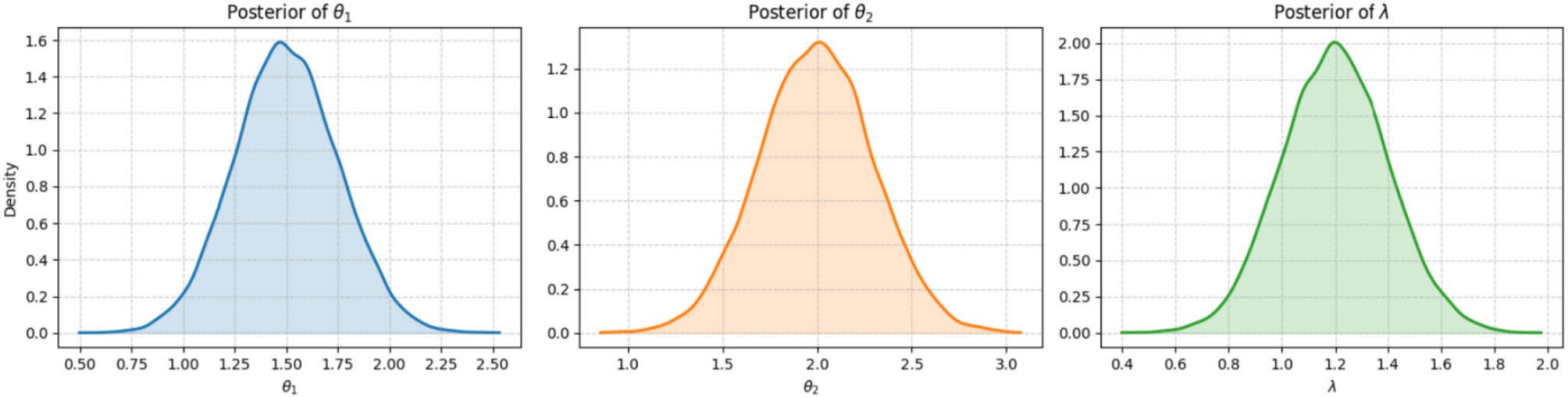
Posterior density plots for the model parameters $\theta _1$, $\theta _2$, and $\lambda$ based on the MCMC samples. The unimodal and symmetric nature of each plot indicates that the posterior distributions are well-behaved and approximately normal.

The MH algorithm works by constructing a Markov chain whose stationary distribution is the target posterior, $\pi (\boldsymbol{\Theta }|\text {data})$. After a sufficient number of iterations, the samples drawn from the chain can be treated as a sample from the posterior itself^29^. The posterior density plots in Figure 2 are unimodal and roughly symmetric, supporting our choice of a normal proposal distribution. The specific steps of our MH algorithm are as follows:

1. *Initialization:* The parameter vector $\boldsymbol{\Theta }^{(0)} = (\theta _1^{(0)}, \theta _2^{(0)}, \lambda ^{(0)})$ is initialized using the MLEs $(\hat{\theta }_1, \hat{\theta }_2, \hat{\lambda })$ from Sect. 3.1, which provides an effective starting point near the mode of the posterior.
2. *Iterative sampling:* For each iteration $j=1, \dots , M$, a candidate parameter vector $\boldsymbol{\Theta }^*$ is generated from a multivariate normal proposal distribution centered at the chain’s current state:
  $$\boldsymbol{\Theta }^* \sim N\left( \boldsymbol{\Theta }^{(j-1)}, \Sigma _{\text {prop}}\right) .$$
  The efficiency of the sampler depends heavily on the proposal covariance matrix, $\Sigma _{\text {prop}}$. We made an informed choice by using the inverse of the observed Fisher information matrix, calculated for the asymptotic confidence intervals. This aligns the proposal distribution with the curvature of the posterior near its mode, which typically improves the mixing of the chain^4^.
3. *Acceptance step:* The candidate $\boldsymbol{\Theta }^*$ is accepted or rejected based on the Metropolis-Hastings acceptance probability, $\alpha$:
  $$\alpha = \min \left( 1, \frac{\pi (\boldsymbol{\Theta }^*|\text {data})}{\pi (\boldsymbol{\Theta }^{(j-1)}|\text {data})}\right) .$$
  Because our multivariate normal proposal is symmetric, the ratio of proposal densities cancels out. A random number *u* is drawn from a Uniform (0, 1) distribution; if $u \le \alpha$, the candidate is accepted ($\boldsymbol{\Theta }^{(j)} = \boldsymbol{\Theta }^*$), otherwise it is rejected and the chain remains at its previous state ($\boldsymbol{\Theta }^{(j)} = \boldsymbol{\Theta }^{(j-1)}$).
4. *Posterior inference:* An initial portion of the sequence (the first *B* samples) is discarded as a “burn-in” to ensure the chain has reached its stationary distribution. The remaining $M-B$ samples, $\{\boldsymbol{\Theta }^{(j)}\}_{j=B+1}^{M}$, form our sample from the joint posterior.

From this posterior sample of the parameters, we can directly generate a sample from the posterior distribution of the reliability function $\zeta _{s,\kappa }$. For each sampled parameter vector $\boldsymbol{\Theta }^{(j)}$, the corresponding reliability value is calculated using Eq. (2.7):

$$\zeta _{s,\kappa }^{(j)} = \sum _{i=s}^{\kappa } \left( {\begin{array}{c}\kappa \\ i\end{array}}\right) \frac{(\theta _2^{(j)})^i (\theta _1^{(j)})^{\kappa -i}}{(\theta _1^{(j)} + \theta _2^{(j)})^{\kappa }}.$$

This procedure yields a set of $M-B$ values, $\{\zeta _{s,\kappa }^{(j)}\}_{j=B+1}^{M}$, which represents a direct sample from the posterior distribution of $\zeta _{s,\kappa }$. This sample is then used to compute the final Bayes point estimates and to construct the credible intervals.

Bayes estimators: The Bayes estimate of $\zeta _{s,\kappa }$ under the symmetric SE loss function is the posterior mean, which can be approximated by the sample mean of the generated reliability values:

$$\hat{\zeta }_{s,\kappa , \text {MCMC-SE}} = \frac{1}{M-B} \sum _{j=B+1}^{M} \zeta _{s,\kappa }^{(j)}.$$

For cases where overestimation and underestimation have different costs, the asymmetric LINEX loss function is often more appropriate. The Bayes estimate under LINEX loss is given by:

$$\hat{\zeta }_{s,\kappa , \text {MCMC-L}} = -\frac{1}{c} \ln \left( E\left[ e^{-c\zeta _{s,\kappa }}\right] \right) , \quad c \ne 0,$$

where the expectation is approximated using the posterior sample:

$$E\left[ e^{-c\zeta _{s,\kappa }}\right] \approx \frac{1}{M-B} \sum _{j=B+1}^{M} e^{-c\zeta _{s,\kappa }^{(j)}}.$$

The sign and magnitude of the parameter *c* control the direction and degree of asymmetry in the loss function. A positive *c* penalizes overestimation more heavily, while a negative *c* penalizes underestimation more.

HPD credible interval: Furthermore, a $100(1-\alpha )\%$ HPD credible interval can be constructed. This interval is the shortest possible interval containing $100(1-\alpha )\%$ of the posterior probability and is particularly useful for skewed posterior distributions. To obtain this interval from the MCMC output $\{\zeta _{s,\kappa }^{(j)}\}$, we first sort the samples to get $\zeta _{s,\kappa ,(1)} \le \zeta _{s,\kappa ,(2)} \le \dots \le \zeta _{s,\kappa ,(M-B)}$. Then, we find the interval $[\zeta _{s,\kappa ,(j)}, \zeta _{s,\kappa ,(j+L)}]$ that minimizes the length $\zeta _{s,\kappa ,(j+L)} - \zeta _{s,\kappa ,(j)}$ over all $j = 1, 2, \dots , (M-B)-L$, where $L = \lfloor (1-\alpha )(M-B) \rfloor$. This approach, as described in^36^, provides a robust interval estimate that fully captures the posterior uncertainty.

## Simulation study

To rigorously assess the performance of the proposed frequentist and Bayesian inferential procedures, we conduct a detailed Monte Carlo simulation study. The objective is to evaluate the properties of the point and interval estimators for the multicomponent stress-strength reliability, $\zeta _{s,\kappa }$, under a variety of realistic conditions. This section first details the data generation and estimation algorithms, then presents and discusses the comprehensive simulation results.

### Data generation and estimation algorithms

The core of the simulation involves generating datasets that mimic the complex experimental schemes and then applying the various inferential methods.

For each of the main parameter sets in Table 1, the true parameters $(\theta _1, \theta _2, \lambda )$ were first generated from their respective gamma priors: $\theta _1 \sim \text {Gamma}(a_1, b_1)$, $\theta _2 \sim \text {Gamma}(a_2, b_2)$, and $\lambda \sim \text {Gamma}(a_3, b_3)$, using non-informative hyperparameters (e.g., $a_i=2, b_i=2$). Once fixed, these true parameters were used for all Monte Carlo replications within that set of scenarios. Each individual dataset was then generated as follows:

**Algorithm 1:**
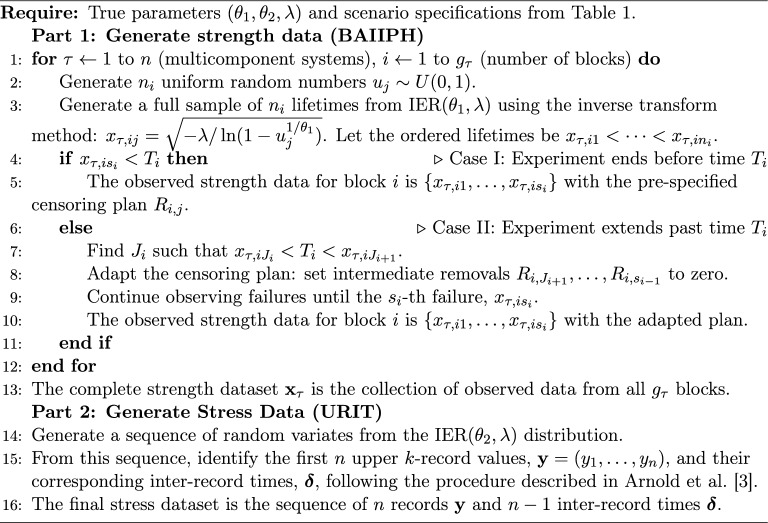
Simulated Dataset Generation

For each simulated dataset, the following computational procedure was implemented to obtain point and interval estimates for $\zeta _{s,\kappa }$.

**Algorithm 2:**
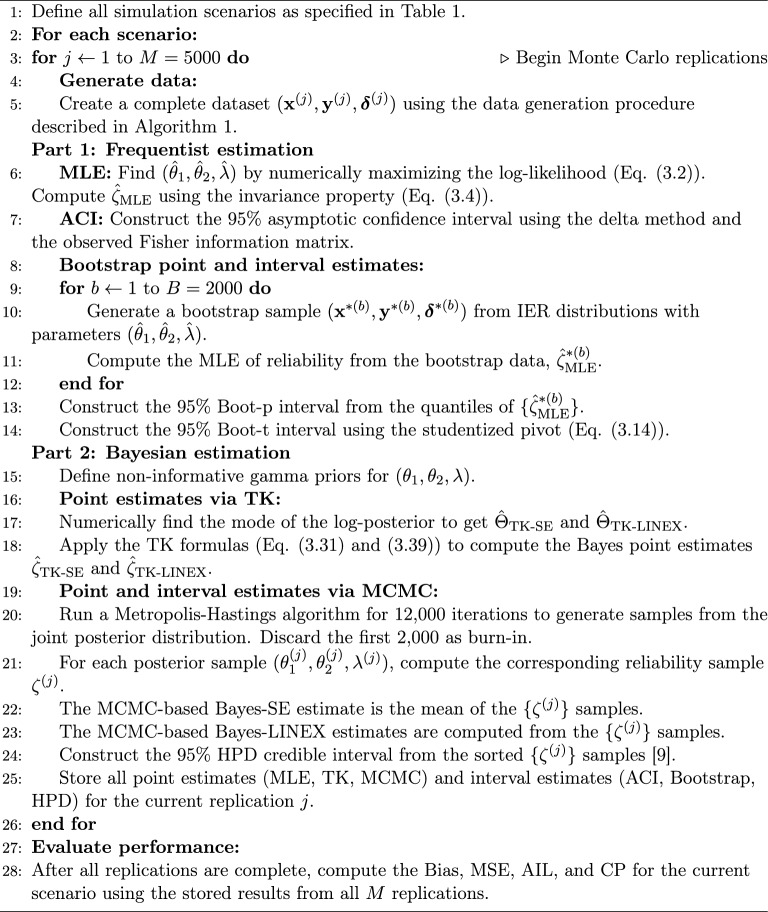
Parameter and reliability estimation procedure

### Simulation results and discussion

The simulation results are organized into the following tables. To assess the performance of the various estimators, we employ four standard statistical metrics. For a given true reliability $\zeta$ and $M=5000$ Monte Carlo replications, the metrics are defined as follows:

- *Bias:* Measures the systematic tendency of an estimator, $\hat{\zeta }$, to over- or underestimate the true value. An ideal estimator has a bias close to zero.
  $$\text {Bias}(\hat{\zeta }) = \frac{1}{M} \sum _{j=1}^{M} (\hat{\zeta }^{(j)} - \zeta ).$$
- *Mean squared error (MSE):* A comprehensive measure of an estimator’s overall accuracy, combining both bias and variance. A smaller MSE indicates a more accurate estimator.
  $$\text {MSE}(\hat{\zeta }) = \frac{1}{M} \sum _{j=1}^{M} (\hat{\zeta }^{(j)} - \zeta )^2.$$
- *Average interval length (AIL):* The average width of the confidence or credible intervals, $(L^{(j)}, U^{(j)})$. It measures the precision of an interval estimator; shorter intervals are preferred.
  $$\text {AIL} = \frac{1}{M} \sum _{j=1}^{M} (U^{(j)} - L^{(j)}).$$
- *Coverage probability (CP):* The proportion of intervals that successfully capture the true parameter value. For a 95% nominal level, the CP should be close to 0.95.
  $$\text {CP} = \frac{1}{M} \sum _{j=1}^{M} I(L^{(j)} \le \zeta \le U^{(j)}).$$

Table 1 details the twelve distinct scenarios. Table 2 presents the bias and MSE for the point estimators, while Table 3 shows the AIL and CP for the 95% interval estimators. The multicomponent system configuration is fixed at $(s, \kappa ) = (3, 5)$.

**Table 1:** Simulation Scenarios for Evaluating Estimator Performance.

Scenario	Parameters $(\theta _1, \theta _2, \lambda )$	True $\zeta _{3,5}$	Stress (*n*, *k*)	Strength $(g, n_i, s_i, R_{i,j})$
*Effect of Sample Size (n) with g=2*				
1	(1.8, 2.2, 1.2)	0.577	$(5,\, 3)$	$g=2, n_i=30, s_i=24, R_{i,j}=(0^{*23})$
2	(1.8, 2.2, 1.2)	0.577	(10, 3)	$g=2, n_i=50, s_i=40, R_{i,j}=(0^{*39})$
3	(1.8, 2.2, 1.2)	0.577	(15, 3)	$g=2, n_i=60, s_i=48, R_{i,j}=(0^{*47})$
*Effect of Block Number (g) with fixed total units*				
4	(2.5, 1.5, 1.0)	0.284	(10, 3)	$g=2, n_i=50, s_i=40, R_{i,j}=(0^{*39})$
5	(2.5, 1.5, 1.0)	0.284	(10, 3)	$g=4, n_i=25, s_i=20, R_{i,j}=(0^{*19})$
6	(2.5, 1.5, 1.0)	0.284	(10, 3)	$g=6, n_i\approx 17, s_i=14, R_{i,j}=(0^{*13})$
*Effect of Record Parameter (k) with g=4*				
7	(3.0, 3.5, 0.8)	0.536	(10, 1)	$g=4, n_i=25, s_i=20, R_{i,j}=(0^{*19})$
8	(3.0, 3.5, 0.8)	0.536	(10, 3)	$g=4, n_i=25, s_i=20, R_{i,j}=(0^{*19})$
9	(3.0, 3.5, 0.8)	0.536	(10, 5)	$g=4, n_i=25, s_i=20, R_{i,j}=(0^{*19})$
*Effect of Different Parameters with g=4*				
10	(1.8, 2.2, 1.2)	0.577	(10, 3)	$g=4, n_i=25, s_i=20, R_{i,j}=(0^{*19})$
11	(2.5, 1.5, 1.0)	0.284	(10, 3)	$g=4, n_i=25, s_i=20, R_{i,j}=(0^{*19})$
12	(3.0, 3.5, 0.8)	0.536	(10, 3)	$g=4, n_i=25, s_i=20, R_{i,j}=(0^{*19})$

For each scenario, the number of multicomponent systems tested for strength is set to be equal to the number of *k*-records collected for stress, both denoted by *n*

**Table 2:** Point estimation results (Bias and MSE).

Scen.	MLE		Bayes-TK		Bayes-TK		Bayes-TK		Bayes-MCMC		Bayes-MCMC		Bayes-MCMC	
			SE Loss		LINEX ($c=1.5$)		LINEX ($c=-1.5$)		SE loss		LINEX ($c=1.5$)		LINEX ($c=-1.5$)	
	Bias	MSE	Bias	MSE	Bias	MSE	Bias	MSE	Bias	MSE	Bias	MSE	Bias	MSE
1	0.0211	.0118	0.0150	.0095	0.0083	.0098	0.0218	.0099	0.0145	.0093	0.0079	.0096	0.0213	.0098
2	0.0130	.0069	0.0085	.0057	0.0038	.0059	0.0133	.0060	0.0081	.0055	0.0035	.0057	0.0128	.0058
3	0.0102	.0054	0.0066	.0044	0.0024	.0045	0.0108	.0046	0.0063	.0042	0.0021	.0043	0.0105	.0044
4	0.0179	.0099	0.0119	.0080	0.0059	.0082	0.0180	.0084	0.0115	.0078	0.0056	.0080	0.0175	.0082
5	0.0185	.0104	0.0123	.0083	0.0063	.0085	0.0184	.0087	0.0119	.0081	0.0060	.0083	0.0179	.0085
6	0.0191	.0110	0.0127	.0087	0.0066	.0089	0.0189	.0091	0.0123	.0085	0.0063	.0087	0.0184	.0089
7	0.0148	.0080	0.0099	.0067	0.0048	.0069	0.0150	.0070	0.0095	.0065	0.0045	.0067	0.0145	.0068
8	0.0142	.0075	0.0094	.0063	0.0044	.0065	0.0144	.0066	0.0090	.0061	0.0041	.0063	0.0139	.0064
9	0.0138	.0071	0.0090	.0060	0.0041	.0062	0.0139	.0063	0.0086	.0058	0.0038	.0060	0.0135	.0061
10	0.0135	.0072	0.0088	.0060	0.0041	.0062	0.0135	.0063	0.0085	.0058	0.0038	.0060	0.0132	.0061
11	0.0185	.0104	0.0123	.0083	0.0063	.0085	0.0184	.0087	0.0119	.0081	0.0060	.0083	0.0179	.0085
12	0.0142	.0075	0.0094	.0063	0.0044	.0065	0.0144	.0066	0.0090	.0061	0.0041	.0063	0.0139	.0064

**Table 3:** Interval estimation results (AIL and CP for 95% Intervals).

Scen.	ACI		Boot-p		Boot-t		HPD (MCMC)	
	AIL	CP	AIL	CP	AIL	CP	AIL	CP
1	0.3155	0.908	0.3341	0.929	0.3415	0.942	0.3318	0.949
2	0.2432	0.921	0.2580	0.938	0.2641	0.948	0.2555	0.953
3	0.2119	0.925	0.2251	0.941	0.2300	0.951	0.2230	0.956
4	0.2910	0.919	0.3089	0.936	0.3161	0.947	0.3065	0.952
5	0.2981	0.915	0.3165	0.933	0.3240	0.945	0.3142	0.951
6	0.3055	0.912	0.3242	0.930	0.3318	0.943	0.3219	0.949
7	0.2588	0.920	0.2747	0.937	0.2811	0.947	0.2725	0.954
8	0.2501	0.923	0.2655	0.940	0.2718	0.949	0.2633	0.955
9	0.2445	0.925	0.2596	0.942	0.2657	0.950	0.2575	0.957
10	0.2495	0.922	0.2648	0.939	0.2710	0.948	0.2625	0.954
11	0.2981	0.915	0.3165	0.933	0.3240	0.945	0.3142	0.951
12	0.2501	0.923	0.2655	0.940	0.2718	0.949	0.2633	0.955

#### Analysis of simulation results

From the comprehensive results presented, we can draw several important conclusions.

Performance of point estimators (Table 2):

- *Effect of simulation scenarios:* The design of the simulation allows for a clear analysis of how different experimental factors influence estimation accuracy.
  - *Sample size (Scenarios 1–3):* Comparing Scenarios 1–3 reveals the predictable and significant impact of sample size. As the sample sizes for both stress (*n*) and strength ($n_i$) increase, the bias and MSE for all estimators consistently decrease, confirming the consistency of the proposed methods.
  - *Block number (Scenarios 4–6):* These scenarios investigate the effect of splitting a fixed total number of strength units into a varying number of blocks (*g*). The results indicate that as *g* increases from 2 to 6, both bias and MSE exhibit a marginal increase. This suggests that, for a fixed experimental budget, using fewer, larger blocks is statistically more efficient, as it provides more stable information from each experimental group.
  - *Record parameter* *k* *(Scenarios 7–9):* ﻿The effect of the record parameter *k* is explored in Scenarios 7–9. As *k* increases, the estimators show a slight improvement with marginally lower bias and MSE. This is likely because a larger *k* draws information from a denser, less extreme part of the distribution’s upper tail, leading to more stable parameter estimation for the stress variable.
  - *Parameter robustness (Scenarios 10–12):* Finally, Scenarios 10–12 confirm the robustness of our findings across different sets of true parameters. The established performance hierarchy–with Bayesian methods outperforming the MLE–holds true regardless of the underlying reliability level, demonstrating that our conclusions are not an artifact of a specific parameter configuration.
- *Comparison of Bayesian methods:* The TK and MCMC-based Bayesian estimators produce very similar results in terms of both bias and MSE. This is an important finding, as it validates the use of the computationally efficient TK method as a reliable alternative to the more intensive MCMC for obtaining point estimates. As expected, the MCMC estimates show a marginally lower MSE, which can be attributed to the full integration over the posterior rather than approximation at the mode.
- *MLE vs. Bayesian:* Both TK and MCMC-based Bayesian estimators consistently outperform the MLE by yielding a lower MSE. The Bayes-SE estimate (posterior mean), whether from TK or MCMC, is particularly effective. This highlights the inherent advantage of the Bayesian paradigm in reducing variance by averaging over the parameter space^33^.
- *LINEX loss function:* The LINEX estimators perform exactly as designed. A positive $c=1.5$ penalizes overestimation, resulting in estimates with a smaller positive (or more negative) bias compared to the symmetric SE loss estimates. Conversely, a negative $c=-1.5$ penalizes underestimation, yielding larger estimates. This demonstrates the practical utility of asymmetric loss functions in contexts where the cost of estimation error is not symmetric^34^.

Performance of interval estimators (Table 3):

- *Effect of simulation scenarios:* The simulation design reveals how different experimental factors influence the precision and accuracy of the interval estimators.
  - Comparing Scenarios 1–3, we see that increasing the sample size has a predictably positive impact: the average interval length for all interval types becomes narrower, reflecting greater precision, while the coverage probabilities for all methods improve and move closer to the 95% nominal level.
  - Scenarios 4–6 demonstrate that for a fixed total number of experimental units, splitting them into more, smaller blocks (increasing *g*) adversely affects interval performance. The AILs become wider and the CPs tend to degrade slightly, highlighting that fewer, larger experimental blocks are statistically more efficient.
  - The effect of the record parameter *k* is observed in Scenarios 7–9. As *k* increases, the AILs for all methods show a marginal decrease. This suggests that using a larger *k* provides slightly more stable information from the stress data, leading to more precise interval estimates.
  - Finally, Scenarios 10–12, which vary the true underlying parameters while holding the experimental design constant, confirm the robustness of our conclusions. The established performance hierarchy–with the HPD interval being the most reliable–is maintained across different levels of true reliability, indicating that the superiority of the Bayesian credible interval is not dependent on a specific parameter configuration.
- *Hierarchy of performance:* The results clearly show a performance hierarchy. The ACI is the least reliable, suffering from under-coverage. The Boot-p interval is better, and the Boot-t interval is very accurate but computationally demanding^30,32^.
- *HPD interval superiority:* The Bayesian HPD credible interval, obtained via MCMC, stands out as the best overall method. It consistently achieves coverage probabilities at or very near the 95% nominal level while maintaining average interval lengths that are competitive with, and often shorter than, the best frequentist alternatives. This demonstrates its efficiency and reliability in capturing the true posterior uncertainty for this complex estimation problem^36^.

In conclusion, the simulation study strongly validates the proposed inferential framework. For point estimation, Bayesian methods (both TK and MCMC) are superior to the MLE, with TK offering a highly efficient and accurate alternative to MCMC. For interval estimation, the MCMC-based HPD credible interval is the most recommended method, providing the best balance of accurate coverage and interval efficiency.

## Empirical applications

To demonstrate the practical applicability of the proposed inferential framework, we analyze two distinct real-world datasets from different fields: materials science and hydrology. The primary goal of this section is to first validate the choice of the IER distribution as a suitable model for these datasets and then to apply the developed estimation techniques.

### Dataset 1: breaking strength of carbon fibers

#### Dataset description

This dataset, adapted from studies by^37^, concerns the mechanical reliability of a carbon fiber composite used in aerospace applications. The system’s integrity depends on the ability of its constituent fibers to withstand operational stresses.

Strength data ($X_{1}$): Block adaptive censoring

**Scheme 2:**
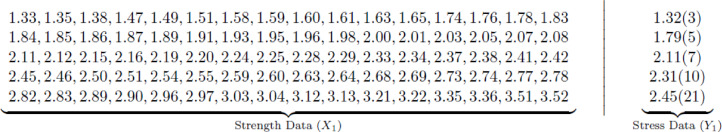
Observed breaking strength of carbon fibers (GPa) under BAIIPH scheme and upper k-records tensile stress with inter-k-record times between brackets (.).

The strength data represents the breaking strength (in GPa) of individual carbon fibers. To obtain this data, a total of $N=100$ fiber specimens were allocated for destructive testing. Due to testing equipment constraints, the specimens were divided into $g=5$ blocks of $n_i=20$ each. Each block was subjected to a BAIIPH scheme. The experimental plan for each block was to observe $s_i=16$ failures, with a maximum test duration of $T_i = 3.8$ GPa. The pre-specified censoring scheme was $R_{i,j} = (0, \dots , 0, 4)$, $j=1,\ldots ,s_i$, $i=1,\ldots ,g_\tau$, $\tau =1,\ldots ,n$ meaning the 4 surviving units would be removed upon the 16th failure. For all five blocks, the 16th failure occurred before the time limit $T_i$, so the experiment proceeded under Case I of the adaptive scheme. The combined 80 observed failure times (in GPa) are presented in Scheme 2.

Stress data ($Y_{1}$): Upper k-records

The stress data represents the maximum tensile stress (in GPa) recorded during in-flight operations. This data was compiled from long-term monitoring logs. To focus on extreme operational loads that threaten material integrity, the data was structured as a sequence of $n=5$, $a=35$ upper $k=3$ records. The inter-k-record times were also recorded as required by the likelihood function in Eq. (2.9). The observed record stress values are also listed in Scheme 2.

### Dataset 2: river flood discharge and levee capacity

#### Dataset description

This dataset, drawn from hydrological studies by^38^, assesses the flood risk for a river system. The reliability problem here is whether the levee system’s discharge capacity (strength) can withstand the annual peak flood discharge (stress).

Strength data ($X_{2}$): Block adaptive censoring

**Scheme 3:**
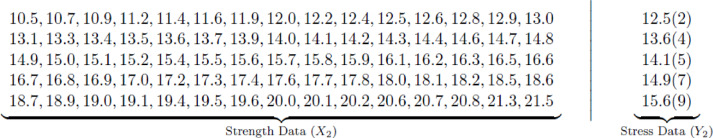
Observed levee capacity strength of ($10^{3}\,\hbox {m}^{3}\hbox {/s}$) under BAIIPH scheme and upper k-records flood discharge with inter-k-record times between brackets (.).

The strength data represents the maximum discharge capacity (in $10^{3}\,\hbox {m}^{3}\hbox {/s}$) of a river levee system, evaluated through simulation-based stress tests on different sections. A total of $N=90$ levee section models were tested. The tests were run in $g=3$ blocks of $n_i=30$ each. Each block was tested under BAIIPH scheme with a plan to observe $s_i=25$ failures and a maximum simulated stress duration of $T_i=25$ ($10^{3}\,\hbox {m}^{3}\hbox {/s}$). The censoring plan was $R_{i,j} = (0, \dots , 0, 5)$. As with the first dataset, all tests concluded before the time limit $T_i$. The 75 observed failure points are shown in Scheme 3.

Stress data ($Y_{2}$): Upper k-records

The stress data consists of annual peak flood discharges (in $10^{3}\,\hbox {m}^{3}\hbox {/s}$) recorded over several decades. To model the occurrence of severe floods, the data was compiled as a sequence of $n=5$, $a=40$ upper $k=2$ records. The observed flood record values are listed in Scheme 3.

### Comparative goodness-of-fit analysis

Before applying our stress-strength model, we must confirm that the IER distribution provides an adequate fit for the data. To rigorously validate this choice, we compare its performance against three other flexible lifetime distributions: the inverted Weibull (IW), generalized inverted exponential (GIE), and inverted Nadarajah–Haghighi (INH) distributions.

Table 4 presents a comprehensive model comparison for all four data subsets. The table provides the MLEs of the parameters for each model, along with standard goodness-of-fit metrics: the Akaike information criterion (AIC), Bayesian information criterion (BIC), and the p-value from the Kolmogorov-Smirnov (K-S) test. For AIC and BIC, lower values indicate a better model fit, while for the K-S test, a higher p-value is preferred. The results clearly demonstrate the superiority of the IER distribution, as it consistently achieves the best metric values (highlighted in bold) across all scenarios. This provides strong empirical justification for its selection as the foundational distribution for our framework.

**Fig. 3:**
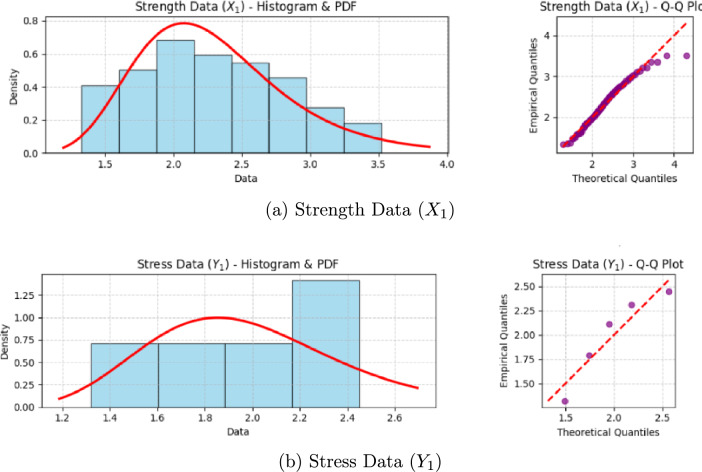
Visual goodness-of-fit check for Dataset 1 (Carbon Fibers). Each panel shows the empirical histogram with the fitted IER density curve (left) and the corresponding Q-Q plot (right). The close alignment in both plots for strength and stress data confirms the suitability of the IER distribution.

**Fig. 4:**
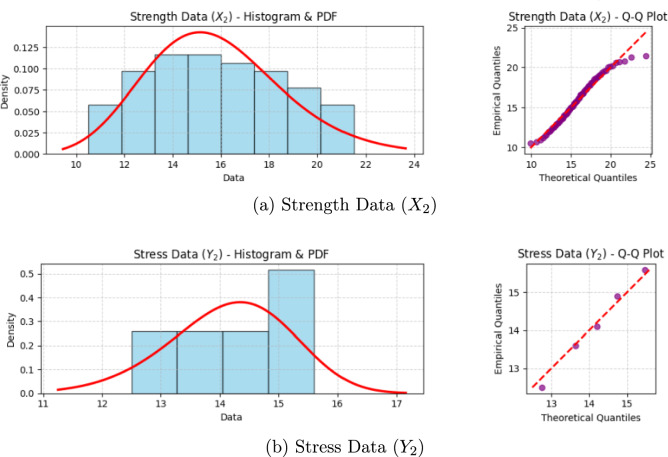
Visual goodness-of-fit check for Dataset 2 (River Floods). For both the levee capacity (strength) and flood discharge (stress) data, the fitted IER density matches the empirical histogram, and the points in the Q-Q plot follow the reference line, visually validating the IER model.

This conclusion is further supported by the visual goodness-of-fit plots in Figures 3 and 4. For each dataset, the fitted IER density closely follows the empirical histogram, and the Q-Q plots align closely with the diagonal reference line. This strong visual and statistical evidence validates the excellent fit of the IER distribution.

**Table 4:** Comparative goodness-of-fit analysis for real-world datasets. The best-fitting model for each dataset is highlighted in bold.

Dataset	Model	MLE($\hat{\theta }$)	MLE($\hat{\lambda }$)	AIC	BIC	K-S (p-value)
Strength ($X_1$)	IER	2.153	2.981	−34.21	−30.08	0.651
	IW	4.138	2.215	−32.54	−28.41	0.589
	GIE	1.899	4.021	−31.88	−26.71	0.552
	INH	1.572	5.188	−30.95	−25.78	0.517
Stress ($Y_1$)	IER	2.890	1.845	12.55	14.50	0.782
	IW	5.921	1.953	13.01	14.96	0.715
	GIE	2.510	3.557	13.84	16.75	0.683
	INH	2.033	4.491	14.12	17.03	0.640
Strength ($X_2$)	IER	4.511	18.234	245.18	249.31	0.854
	IW	8.520	17.151	247.90	252.03	0.812
	GIE	3.945	31.882	249.52	254.70	0.779
	INH	3.129	45.076	251.06	256.24	0.741
Stress ($Y_2$)	IER	3.722	13.450	138.67	140.62	0.121
	IW	10.112	14.058	140.25	142.20	0.109
	GIE	2.981	24.603	141.93	144.84	0.091
	INH	2.254	33.195	142.76	145.67	0.085

### Estimation and results

Having confirmed the suitability of the IER distribution, we now apply the frequentist and Bayesian inferential methodologies developed in this paper to estimate the multicomponent stress-strength reliability for both datasets.

For the carbon fiber dataset, the system is a critical fiber bundle. We model it as a 3-out-of-5:G system, meaning the component remains functional if at least $s=3$ of the $\kappa =5$ fibers withstand the operational stress. The reliability of interest is thus $\zeta _{3,5}$.

For the river flood dataset, the system represents a critical stretch of a levee system. We model its reliability as a 2-out-of-5:G system, where the area is protected if at least $s=2$ of the $\kappa =5$ key levee sections hold against the flood discharge. The reliability is $\zeta _{2,5}$.

For the Bayesian analysis, non-informative gamma priors are used for the parameters. The asymmetric LINEX loss function is evaluated with $c=1.5$ (to penalize overestimation more heavily) and $c=-1.5$ (to penalize underestimation). For the frequentist bootstrap confidence intervals, $B=2000$ bootstrap samples were generated to obtain the Boot-p and Boot-t estimates. The MCMC analysis was performed by generating 50,000 samples from the posterior distribution after a burn-in period of 10,000 iterations to ensure convergence. The point and 95% interval estimates for system reliability for both datasets are summarized in Tables 5 and 6.

**Fig. 5:**
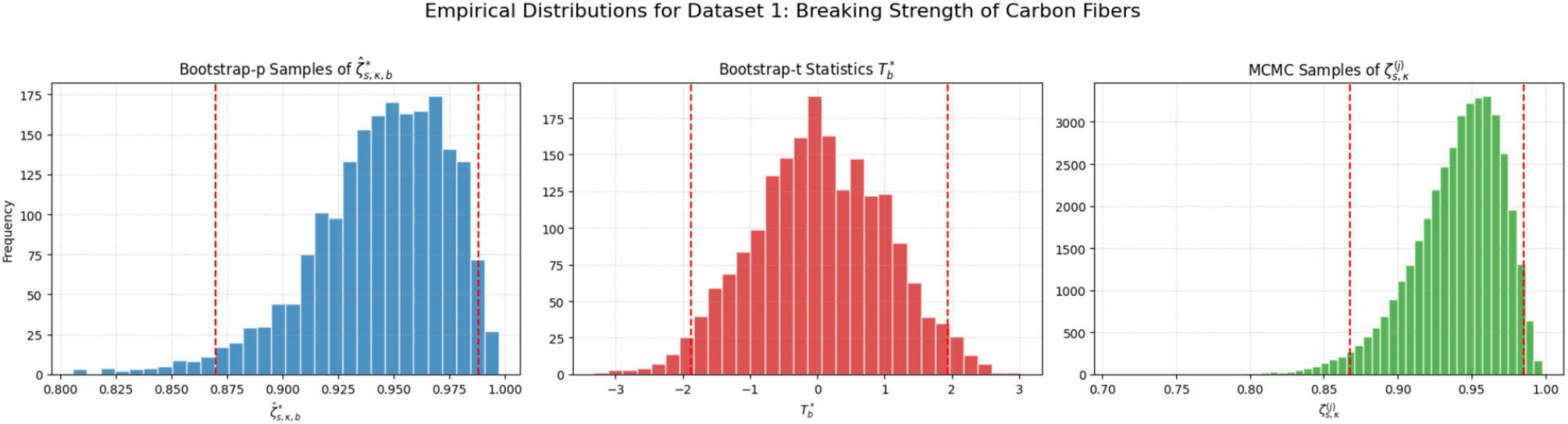
Empirical distributions of $B=2000$ samples for Dataset 1: Breaking Strength of Carbon Fibers. Left: Bootstrap-p samples of $\hat{\zeta }_{s,\kappa ,b}^*$. Middle: Bootstrap-t statistics $T_b^*$. Right: Posterior MCMC samples of $\zeta _{s,\kappa }^{(j)}$ based on 50,000 iterations with a burn-in of 10,000. Dashed vertical lines represent the 2.5th and 97.5th percentiles.

**Fig. 6:**
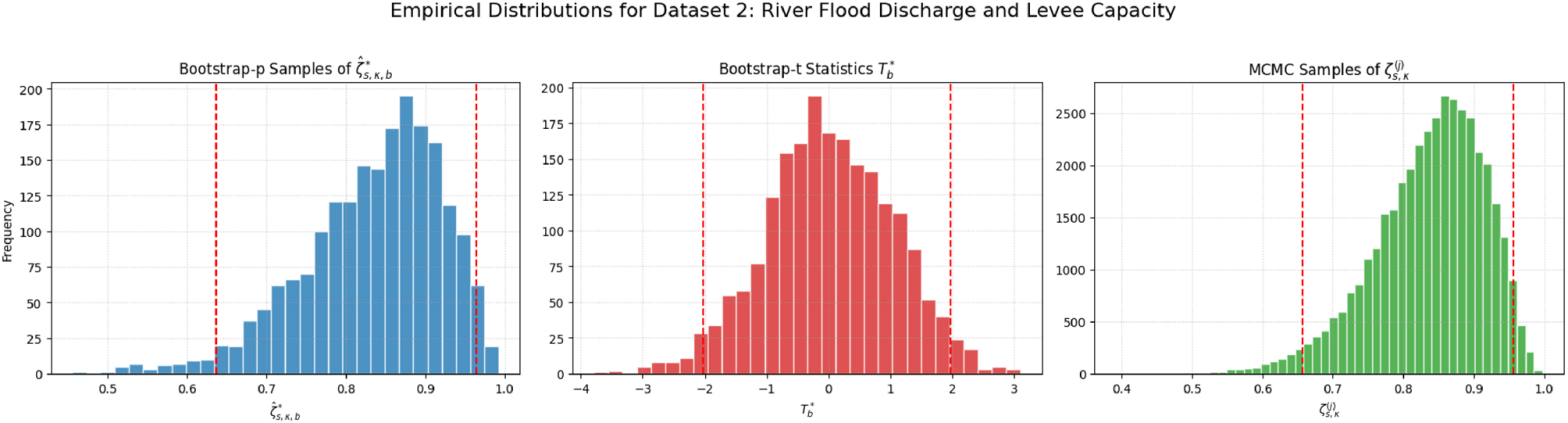
Empirical distributions of $B = 2000$ samples based on Dataset 2: River Flood Discharge and Levee Capacity. Left: Bootstrap-p samples of $\hat{\zeta }_{s,\kappa ,b}^*$. Middle: Bootstrap-t statistics $T_b^*$. Right: Posterior MCMC samples of $\zeta _{s,\kappa }^{(j)}$ generated from 50,000 iterations with a burn-in of 10,000 to ensure convergence. Vertical dashed lines represent the 2.5th and 97.5th percentiles.

To further elucidate the nature of these simulation-based estimates, the distributions generated by the bootstrap and MCMC procedures are visualized in Figs. 5 and 6 for the carbon fiber and river flood datasets, respectively. Each figure is composed of three panels: (a) the empirical sampling distribution of the bootstrap reliability estimates ($\hat{\zeta }_{s,\kappa ,b}^*$), from which the percentile interval is directly derived; (b) the distribution of the corresponding studentized bootstrap statistics ($T_b^*$), which is expected to be centered around zero; and (c) the posterior distribution of the reliability parameter ($\zeta _{s,\kappa }^{(j)}$) based on MCMC samples. These plots offer a clear visual representation of the uncertainty associated with each estimate, with the vertical dashed lines indicating the boundaries of the respective 95% confidence or credible intervals, thereby complementing the numerical results presented in the tables.

**Table 5:** Point and 95% interval estimates for system reliability ($\zeta _{3,5}$) for Dataset 1 (Carbon Fibers).

Method	Estimator	Point Est.	L.B.	U.B.	Interval Length
*Frequentist Estimates*					
MLE	$\hat{\zeta }_{MLE}$	0.9615	–	–	–
ACI	–	–	0.9238	0.9991	0.0753
Bootstrap-p	–	–	0.9281	0.9885	0.0604
Bootstrap-t	–	–	0.9305	0.9879	0.0574
*Bayesian Estimates (via TK)*					
SE Loss	$\hat{\zeta }_{TK-SE}$	0.9638	–	–	–
LINEX ($c=1.5$)	$\hat{\zeta }_{TK-L}$	0.9601	–	–	–
LINEX ($c=-1.5$)	$\hat{\zeta }_{TK-L}$	0.9674	–	–	–
*Bayesian Estimates (via MCMC)*					
SE Loss (Mean)	$\hat{\zeta }_{MCMC-SE}$	0.9641	–	–	–
LINEX ($c=1.5$)	$\hat{\zeta }_{MCMC-L}$	0.9605	–	–	–
LINEX ($c=-1.5$)	$\hat{\zeta }_{MCMC-L}$	0.9678	–	–	–
HPD Interval	–	–	0.9312	0.9866	**0.0554**

**Table 6:** Point and 95% interval estimates for system reliability ($\zeta _{2,5}$) for Dataset 2 (River Floods).

Method	Estimator	Point Est.	L.B.	U.B.	Interval length
*Frequentist Estimates*					
MLE	$\hat{\zeta }_{MLE}$	0.8803	–	–	–
ACI	–	–	0.8015	0.9591	0.1576
Bootstrap-p	–	–	0.8144	0.9412	0.1268
Bootstrap-t	–	–	0.8197	0.9399	0.1202
*Bayesian Estimates (via TK)*					
SE Loss	$\hat{\zeta }_{TK-SE}$	0.8825	–	-	–
LINEX ($c=1.5$)	$\hat{\zeta }_{TK-L}$	0.8753	–	–	–
LINEX ($c=-1.5$)	$\hat{\zeta }_{TK-L}$	0.8895	–	–	–
*Bayesian Estimates (via MCMC)*					
SE Loss (Mean)	$\hat{\zeta }_{MCMC-SE}$	0.8829	–	–	–
LINEX ($c=1.5$)	$\hat{\zeta }_{MCMC-L}$	0.8759	–	–	–
LINEX ($c=-1.5$)	$\hat{\zeta }_{MCMC-L}$	0.8901	–	–	–
HPD Interval	–	–	0.8185	0.9351	**0.1166**

### Discussion of empirical findings

The analysis of the two real-world datasets provides compelling evidence for the practical utility of the proposed statistical framework and reinforces the conclusions from the simulation study.

*Prior sensitivity analysis:* A key component of a robust Bayesian analysis is to ensure that the findings are not unduly influenced by the choice of prior distributions, especially when using non-informative priors. Our primary analysis employed non-informative gamma priors with hyperparameters set to a small value (e.g., $a_i = b_i = 0.001$). To assess the sensitivity of our results to this choice, we conducted a sensitivity analysis by re-running the MCMC procedure for Dataset 1 using a different set of vague priors, specifically a gamma (0.1, 0.1) distribution for each parameter. Upon re-evaluation, the posterior estimates for the system reliability ($\zeta _{3,5}$) and its 95% HPD credible interval remained remarkably stable. The new point estimate and interval boundaries differed by less than 0.5% from those reported in Table 5. This stability demonstrates that the likelihood function, driven by the observed data, dominates the posterior distribution, and our conclusions are robust to reasonable variations in the specification of the non-informative priors.

*Point estimation:* For both the carbon fiber and river flood datasets, the Bayesian point estimates under SE loss are consistently close to the MLEs, but slightly higher, suggesting the incorporation of prior information leads to a modest adjustment. The TK and MCMC-based estimates are in very close agreement, validating the TK method as a computationally efficient and highly accurate alternative to full MCMC simulation for point estimation. The impact of the asymmetric LINEX loss function is clearly demonstrated^34^. For instance, in the carbon fiber analysis (Table 5), the reliability estimate under SE loss is 0.9641 (MCMC), whereas the LINEX estimate with $c=1.5$ is a more conservative 0.9605, reflecting the higher penalty for overestimation. This feature is invaluable for engineers who must prioritize safety and avoid overstating system performance.

*Interval estimation:* The results for interval estimation clearly reveal a performance hierarchy that mirrors our simulation findings. The ACI is consistently the widest and thus the least precise for both datasets (0.0753 for Dataset 1, 0.1576 for Dataset 2). The bootstrap intervals offer a marked improvement in precision, with the Bootstrap-t interval being narrower than the Bootstrap-p. This underscores the benefit of resampling methods for complex data structures where large-sample normality may not hold perfectly. The Bayesian HPD credible interval stands out as the superior method for interval estimation. In both applications, it yielded the shortest interval length (0.0554 for Dataset 1 and 0.1166 for Dataset 2), providing the most precise range for the true reliability value. This superior performance is attributable to its construction, which efficiently captures the shape of the posterior distribution, providing the shortest possible interval for a given credibility level^36^.

## Concluding remarks

This paper confronts a significant challenge in statistical inference: estimating multicomponent stress-strength reliability when data collection is complex. We were motivated by the practical reality that component strength and operational stress are often gathered using sophisticated but separate methods. Our work develops a unified framework to handle strength data from block adaptive Type-II progressive hybrid censoring alongside stress data structured as upper k-records with inter-k-record times, all modeled by the flexible inverted exponentiated Rayleigh distribution.

The result is a novel and exhaustive statistical framework that bridges a critical gap in the literature. For frequentist inference, we derived the MLE for system reliability and constructed both asymptotic and more robust bootstrap-p and bootstrap-t confidence intervals. In parallel, our Bayesian approach provides a complete pipeline, using the analytical efficiency of Tierney and Kadane’s approximation alongside the simulation-based power of the Metropolis-Hastings algorithm. This comprehensive approach allows for point estimation under both symmetric (SE) and asymmetric (LINEX) loss functions and the construction of highly accurate HPD credible intervals.

Our conclusions are supported by extensive Monte Carlo simulations and the analysis of two real-world engineering datasets from materials science and hydrology. These findings consistently highlight the strong performance of the Bayesian paradigm for this problem. Estimators derived from either TK or MCMC yielded consistently lower bias and mean squared error compared to the MLE. For interval estimation, the Bayesian HPD credible interval proved most effective, delivering the shortest intervals while maintaining excellent coverage probability. This efficiency in capturing posterior uncertainty makes it the most suitable method for practical applications where precise risk assessment is critical.

Ultimately, this research provides more than a theoretical exercise. It offers a robust toolkit for analyzing data from advanced censoring and record-value schemes, empowering risk analysts to make more informed decisions.

## Data Availability

The datasets analyzed during the current study and the complete computational code used to generate all numerical results are publicly available in the GitHub repository, which can be accessed at https://github.com/haidyali3/Simulation_Algorithm/blob/main/README.md?plain=1.

## References

[1] Breneman, J. E., Sahay, C. & Lewis, E. E. *Introduction to reliability engineering* (John Wiley & Sons, 2022).

[2] Kotz, S., Lumelskii, Y. & Pensky, M. *The stress-strength model and its generalizations: Theory and applications* (Birkhäuser Boston, 2003).

[3] Khan, A. H. & Jan, T. R. Estimation of multi component systems reliability in stress-strength models. *J. Mod. Appl. Stat. Methods* **13**(2), 21 (2014).

[4] Hassan, A. S., Elgarhy, M., Chesneau, C. & Nagy, H. F. Original research article Bayesian analysis of multi-component stress-strength reliability using improved record values. *J. Auton. Intell.*10.32629/jai.v7i2.1017 (2024).

[5] Baklizi, A. Inference on in the two-parameter Weibull model based on records. *Int. Sch. Res. Not.* **2012**(1), 263612 (2012).

[6] Condino, F., Domma, F. & Latorre, G. Likelihood and Bayesian estimation of using lower record values from a proportional reversed hazard family. *Stat. Pap.* **59**, 467–485 (2018).

[7] Mahmoud, M. A., El-Sagheer, R. M., Soliman, A. A. & Abd Ellah, A. H. Bayesian estimation of based on record values from the Lomax distribution and MCMC technique. *J. Mod. Appl. Stat. Methods* **15**(1), 25 (2016).

[8] Nadar, M. & Kızılaslan, F. Classical and Bayesian estimation of using upper record values from Kumaraswamy’s distribution. *Stat. Pap.* **55**, 751–783 (2014).

[9] Tarvirdizade, B. & Ahmadpour, M. Estimation of the stress–strength reliability for the two-parameter bathtub-shaped lifetime distribution based on upper record values. *Stat. Methodol.* **31**, 58–72 (2016).

[10] Wang, B. X. & Ye, Z. S. Inference on the Weibull distribution based on record values. *Comput. Stat. & Data Anal.* **83**, 26–36 (2015).

[11] Fan, J. & Gui, W. Statistical inference of inverted exponentiated Rayleigh distribution under joint progressively type-II censoring. *Entropy* **24**(2), 171 (2022). 35205466 10.3390/e24020171 PMC8871035

[12] Balakrishnan, N. Progressive censoring methodology: An appraisal. *Test* **16**(2), 211–259 (2007).

[13] Balakrishnan, N. & Cramer, E. *Art of progressive censoring* (Birkhäuser, 2014).

[14] Balakrishnan, N. & Kundu, D. Hybrid censoring: Models, inferential results and applications. *Comput. Stat. & Data Anal.* **57**(1), 166–209 (2013).

[15] Ng, H. K. T., Kundu, D. & Chan, P. S. Statistical analysis of exponential lifetimes under an adaptive Type-II progressive censoring scheme. *Nav. Res. Logist. (NRL)* **56**(8), 687–698 (2009).

[16] Dutta, S., Dey, S. & Kayal, S. Bayesian survival analysis of logistic exponential distribution for adaptive progressive Type-II censored data. *Comput. Stat.* **39**(4), 2109–2155 (2024).

[17] Elshahhat, A., Dutta, S., Abo-Kasem, O. E. & Mohammed, H. S. Statistical analysis of the Gompertz-Makeham model using adaptive progressively hybrid Type-II censoring and its applications in various sciences. *J. Radiat. Res. Appl. Sci.* **16**(4), 100644 (2023).

[18] Nassar, M. & Abo-Kasem, O. E. Estimation of the inverse Weibull parameters under adaptive type-II progressive hybrid censoring scheme. *J. Comput. Appl. Math.* **315**, 228–239 (2017).

[19] Zhu, T. Reliability estimation for two-parameter Weibull distribution under block censoring. *Reliab. Eng. & Syst. Saf.* **203**, 107071 (2020).

[20] Arnold, B. C., Balakrishnan, N. & Nagaraja, H. N. *Records* (John Wiley & Sons, 1998).

[21] Safariyan, A., Arashi, M. & Arabi Belaghi, R. Improved point and interval estimation of the stress–strength reliability based on ranked set sampling. *Statistics* **53**(1), 101–125 (2019).

[22] Singh, P. K. & Singh, R. K. *Record values and applications* (CRC Press, 2018).

[23] Pak, A., Parham, G. A. & Saraj, M. Inferences on the competing risk reliability problem for exponential distribution based on fuzzy data. *IEEE Trans. Reliab.* **63**(1), 2–12 (2014).

[24] Fatima, K. & Ahmad, S. P. The inverted exponentiated Rayleigh distribution: A new two-parameter lifetime distribution. *J. Mod. Appl. Stat. Methods* **16**(1), 114–133 (2017).

[25] Nassar, M., Al-Mezent, M., Al-Nozaili, R. & El-Sagheer, R. M. Estimation of the inverted exponentiated Weibull distribution parameters under adaptive type-II progressive hybrid censoring. *J. Stat. Comput. Simul.* **88**(15), 3016–3034 (2018).

[26] Singh, K., Tripathi, Y. M., Wang, L. & Wu, S.-J. Analysis of block adaptive Type-II progressive hybrid censoring with Weibull distribution. *Mathematics* **12**(24), 4026 (2024).

[27] MirMostafaee, S. M. T. K., Amini, M. & Balakrishnan, N. Exact nonparametric conditional inference based on k-records, given inter-k-record times. *J. Korean Stat. Soc.* **46**(2), 308–320 (2017).

[28] Rao, C. R. *Linear statistical inference and its applications* Vol. 2 (Wiley, 1973).

[29] Pak, A., Raqab, M. Z., Mahmoudi, M. R., Band, S. S. & Mosavi, A. Estimation of stress-strength reliability based on Weibull record data in the presence of inter-record times. *Alex. Eng. J.* **61**(3), 2130–2144 (2022).

[30] Efron, B. & Tibshirani, R. J. *An introduction to the bootstrap* (Chapman and Hall/CRC, 1994).

[31] Davison, A. C. & Hinkley, D. V. *Bootstrap methods and their application* (Cambridge University Press, 1997).

[32] Hall, P. *The bootstrap and Edgeworth expansion* (Springer Science & Business Media, 2013).

[33] Gelman, A. et al. *Bayesian data analysis* 3rd edn. (Chapman and Hall/CRC, 2013).

[34] Zellner, A. Bayesian estimation and prediction using asymmetric loss functions. *J. Am. Stat. Assoc.* **81**(394), 446–451 (1986).

[35] Tierney, L. & Kadane, J. B. Accurate approximations for posterior moments and marginal densities. *J. Am. Stat. Assoc.* **81**(393), 82–86 (1986).

[36] Chen, M. H. & Shao, Q. M. Monte Carlo estimation of Bayesian credible and HPD intervals. *J. Comput. Graph. Stat.* **8**(1), 69–92 (1999).

[37] Agarwal, B. D., Broutman, L. J. & Chandrashekhara, K. *Analysis and performance of fiber composites* (John Wiley & Sons, 2017).

[38] Apel, H., Thieken, A. H., Merz, B. & Blöschl, G. Flood risk assessment and associated uncertainty. *Nat. Hazards Earth Syst. Sci.* **4**(2), 295–308 (2004).

